# ﻿Diminishing the taxonomic gap in the neotropical soldierless termites: descriptions of four new genera and a new *Anoplotermes* species (Isoptera, Termitidae, Apicotermitinae)

**DOI:** 10.3897/zookeys.1167.100001

**Published:** 2023-06-22

**Authors:** Tiago F. Carrijo, Daniel Castro, Menglin Wang, Joice P. Constantini, Thomas Bourguignon, Eliana M. Cancello, Yves Roisin, Rudolf H. Scheffrahn

**Affiliations:** 1 Centro de Ciências Naturais e Humanas, Universidade Federal do ABC, Av. dos Estados, 5001, Sta. Terezinha, 09210-580, Santo André, SP, Brazil; 2 Instituto Amazónico de Investigaciones Científicas SINCHI, Avenida Vásquez Cobo Calles 15 y 16, Leticia,; 3 , Colombia; 4 Okinawa Institute of Science and Technology Graduate University, 1919-1 Tancha, Onna-son, Okinawa, 904-0495, Japan; 5 Museu de Zoologia da Universidade de São Paulo, Cx. Postal 42391, CEP 04218–970, São Paulo, SP, Brazil; 6 Faculty of Tropical AgriSciences, Czech University of Life Sciences, Prague, Czech Republic; 7 Evolutionary Biology and Ecology, Université Libre de Bruxelles, Avenue F.D. Roosevelt 50, 1050 Brussels, Belgium; 8 Fort Lauderdale Research and Education Center, Institute for Food and Agricultural Sciences, University of Florida, 3205 College Avenue, Davie, Florida 33314, USA

**Keywords:** Enteric valve armature, Linnean shortfall, mitogenome sequencing, soil-feeder, species distribution, taxonomy

## Abstract

The neotropical Apicotermitinae is a common and widespread clade of mostly soil-feeding soldierless termites. With few exceptions, species of this group were originally assigned to the genus *Anoplotermes* Müller, 1873. The application of internal worker morphology coupled with genetic sequencing has recently shed light on the true diversity of this subfamily. Herein, *Anoplotermessusanae* Scheffrahn, Carrijo & Castro, **sp. nov.** and four new species in four new genera are described: *Hirsutitermeskanzakii* Scheffrahn, Carrijo & Castro, **gen. nov. et sp. nov.**, *Krecekitermesdaironi* Scheffrahn, Carrijo & Castro, **gen. nov. et sp. nov.**, *Mangolditermescurveileum* Scheffrahn, Carrijo & Castro, **gen. nov. et sp. nov.**, and *Ourissotermesgiblinorum* Scheffrahn, Carrijo & Castro, **gen. nov. et sp. nov.** Worker descriptions are based mainly on worker gut morphology, including the enteric valve, while imagoes were described based on external characters. A Bayesian phylogenetic tree of New World Apicotermitinae was constructed using the complete mitogenome to infer genera relationships and corroborate the taxonomic decisions. Distribution maps and a dichotomic key to the known Neotropical Apicotermitinae genera are provided.

## ﻿Introduction

Termites are dominant fauna of tropical ecosystems, having a high abundance, density, and biomass, especially in tropical soils (Fittkau and Klinge 1973; Barros et al. 2004; Menta and Remelli 2020; Demetrio et al. 2021). During their feeding and construction activities, soil-feeding termites provide many ecosystem services (Jouquet et al. 2011; Lavelle et al. 2014; Duran-Bautista et al. 2020b). Termites have a wide diet based on organic matter ranging across the humification spectrum of the feeding substrate (Eggleton 2011). Many authors classified them into functional or feeding groups (De Souza and Brown 1994; Eggleton et al. 1997; Donovan et al. 2001; Eggleton and Tayasu 2001; Davies 2002), the most used of which are those of Donovan et al. (2001), who proposed groups III and IV for ‘true’ soil-feeders, or humus-feeders. This classification, however, shows some limitations for neotropical termites, because the correlation among morphological differences in the enteric valve armature (EVA), mandibles and the humification gradient does not always follows the pattern described (Bourguignon et al. 2011b). Thus, the use of the terms soil- or humus-feeders might be more appropriate for this taxon.

Soil-feeding, the ability to digest intractable organic elements from the soil, is exclusive to the family Termitidae (Eggleton and Tayasu 2001; Bucek et al. 2019). As far as we know, most of the neotropical soldierless termites (Apicotermitinae) are soil-feeders (except for *Ruptitermes*, some species of *Aparatermes*, and *Anoplotermespacificus* – for the last one see Kaiser 1953), and their abundance and species richness can reach up to 50% of all termite records in some assemblages of South America (Carrijo et al. 2009; Bourguignon et al. 2011a; Palin et al. 2011; da Silva et al. 2019; Dahlsjö et al. 2020; Castro et al. 2021).

Identification of termite workers requires examination of gut morphology, especially that of the enteric valve armature (EVA), one of the most important diagnostic characters for them (Scheffrahn 2013; Bourguignon et al. 2016b; Scheffrahn et al. 2017; Castro et al. 2018, 2020). The dissection of the EVA is complicated and requires a trained specialist, imposing difficulties and often limiting identification of soldierless species to morphospecies status in many studies (Sanabria et al. 2016; Casalla and Korb 2019; Duran-Bautista et al. 2020a). Moreover, it has been shown that some recently described species have a widespread distribution (Scheffrahn et al. 2017; Constantini et al. 2018), as in the case of *Rustitermesboteroi* (Castro et al. 2020), and can be indicator species of disturbed habitats (Castro et al. 2021).

Currently, 57 species belonging to 17 genera of soldierless termites are recognized in the Neotropics (Constantino 2022), representing 25% of the world’s Apicotermitinae. However, many species and genera are still to be described in Central and South America (Bourguignon et al. 2013) (for some examples, see the website: https://www.termitediversity.org). Nine of the 17 described genera in the Neotropics are monotypic, and molecular analyses have pointed out that a large number of undescribed genera are monotypic (Bourguignon et al. 2013; Scheffrahn 2019). The Neotropical genera *Anoplotermes* (21 spp.) and *Ruptitermes* (13 spp.) are the most speciose, followed by *Aparatermes* (4 spp.), and five genera with two described species to date (*Compositermes*, *Humutermes*, *Hydrecotermes*, *Patawatermes*, and *Tonsuritermes*). Nevertheless, the genus *Anoplotermes* needs to be taxonomically revised, which will likely lead to the description of new genera and synonymies (Bourguignon et al. 2010, 2016b). The descriptions of new soldierless taxa are paramount to fill what is probably the main taxonomic gap (i.e., Linnean shortfall) in termitology. Filling this gap will facilitate future research on neotropical termites, especially related to soil ecology, where soldierless termites are very abundant and diverse (Bourguignon et al. 2016a; Rodríguez-León et al. 2021).

In this paper, we describe four monotypic soldierless genera and a new *Anoplotermes* species based on the morphology of the worker and imago castes. We also provide a molecular phylogenetic analysis based on the complete mitogenome, including all but two neotropical Apicotermitinae genera. Finally, an illustrated dichotomous key for the worker caste is provided based on the diagnostic characters of the genera published to date.

## ﻿Material and method

The specimens examined in this study were collected and preserved in 75%, 85%, or 92% ethanol and were deposited at the Museu de Zoologia da Universidade de São Paulo, São Paulo, Brazil (**MZUSP**); the Museo Entomológico de la Facultad de Agronomía of the Universidad Nacional de Colombia, Bogotá D.C, Colombia (**UNAB**); the Colección de Artrópodos Terrestres de la Amazonía Colombiana of the SINCHI Institute in Leticia, Amazonas, Colombia (**CATAC**); the University of Florida Termite Collection at Fort Lauderdale Research and Education Center, Davie, Florida, United States (**UFTC**, Scheffrahn 2019); and the Unit of Evolutionary Biology and Ecology, Université Libre de Bruxelles, Brussels, Belgium (**ULB**).

Morphological examination of the enteric valve armature (EVA) and digestive tube was done on the worker caste. The terminology used for the worker digestive tube and mandibles follows Noirot (2001), Sands (1972), and Deligne (1999). We used the term “molar process” proposed by Constantini et al. (2020). The following morphometric characters were measured for workers and/or alates [the numbers in parentheses are in accordance with Roonwal (1970)]: length of head to lateral base of mandibles (5), maximum head width (17), inter-eye (between eye) distance (52), maximum ocellus diameter (55), minimum ocellus diameter (56), maximum diameter of eye (48), minimum diameter of eye with margin (49), pronotum length (65), pronotum width (68), hind tibia and fore tibia lengths (85), width of fore tibia (86), and fore tibia width: length ratio (index 53). Size of individuals (imagoes and workers) are described in reference to other neotropical soldierless termites, and they can be very small, small, medium, or large.

Molecular phylogenetic analyses were performed on a dataset including the four new genera in addition to 15 of the 17 previously described genera of Apicotermitinae from the New World (Fig. 12, Suppl. material 1). Samples of the new taxa used in the phylogeny are highlighted in the examined material with two asterisks (**). The two missing genera were *Echinotermes* and *Amplucrutermes*. We also included *Anoplotermessusanae* sp. nov. and, for the first time, the type of the genus *Anoplotermes*, *A.pacificus* Müller, 1873. We sequenced the mitochondrial genomes of samples of Apicotermitinae preserved in 80% ethanol and stored at room temperature for more than 20 years. Briefly, whole genomic DNA was extracted from entire termite workers (including gut) using the DNeasy Blood & Tissue extraction kit (Qiagen). Libraries were prepared using the NEBNext Ultra II FS DNA Library Preparation Kit (New England Biolabs) and the Unique Dual Indexing Kit (New England Biolabs) without enzymatic fragmentation step and with one-fifteenth of the reagent volumes recommended by the manufacturer. Libraries were paired-end sequenced using the Illumina HiSeq X or Novaseq platforms.

The alignment of the 13 mitochondrial protein-coding genes and the two ribosomal RNAs was performed separately with the Mafft Multiple Alignment plugin (Katoh and Standley 2013) implemented in Geneious v. 9.1 (Biomatters Ltd, Auckland, New Zealand). The alignments were edited by eye. An alignment of 15,024 bp was obtained and partitioned in protein-coding genes and ribosomal RNA. The model GTR+I+G was selected for both partitions by ModelFinder (Kalyaanamoorthy et al. 2017) in the IQ-TREE web server (Nguyen et al. 2015; Trifinopoulos et al. 2016). Phylogenetic analyses were performed by Maximum Likelihood (ML) and Bayesian Inference (BI), with the IQ-TREE and BEAST 1.8.0 (Drummond and Rambaut 2007), respectively. For the BI, we used a Yule speciation process (Gernhard 2008) and a strict molecular clock as priors. Note that these analyses were performed to estimate the relationships among genera of Apicotermitinae rather than divergence time. The analysis was performed with Markov chain Monte Carlo searches (MCMC) conducted for 50 million generations. Convergence and stationarity were assessed with Tracer 1.6 (Suchard et al. 2018), and the tree was visualized using Figtree 1.3.1. Results between analyses were similar, and we preferred the BI for results and discussion, but divergences were pointed in the text.

Distribution maps (Fig. 13) were created using ArcGIS desktop 10.8 (ESRI, Redlands, CA). Microphotographs were taken using a Leica S9i stereomicroscope as multi-layered mounts with an integrated Leica digital camera for workers and imagoes. Worker mandibles and EVA microphotographs from the MZUSP were taken using a Leica ICCM50 W microscope with an integrated camera. Enteric valves from UFTC collection were photographed with a Leica CTR 5500 compound microscope. EVA from CATAC and UNAB were photographed with an Olympus BX53 microscope coupled to an Olympus DP27 digital camera.

## ﻿Results

### ﻿Taxonomy

***Anoplotermes* Müller, 1873**

#### ﻿ Anoplotermes susanae

Taxon classificationAnimaliaIsopteraTermitidae

Scheffrahn, Carrijo & Castro sp. nov.

E77CE35E-0A57-5CB3-9F2B-22F314C58375

https://zoobank.org/3B3007E4-A901-4AC0-B676-B92F5F0A4F3B

##### Material examined.

***Holotype*.** Worker from lot MZUSP 13213** (in a separate vial with the remaining sample).

##### Type locality.

Brazil. Rondônia, Porto Velho, Mutum-Paraná, -9.6375, -65.0567.

##### Type repository.

MZUSP.

***Paratypes*.** Brazil. Rondônia, Porto Velho, Abunã, (-9.6257, -65.4423), 15.MAY.2010, 122 m, TF Carrijo and MM Rocha coll. (MZUSP 17671); Porto Velho, Mutum-Paraná,(-9.4390, -64.8409), 17.SEP.2011, 110 m, TF Carrijo and LR Fernandes coll. (MZUSP 17684); (-9.4406, -64.8497), 26.JUN.2010, 116 m, TF Carrijo and SP Rosa coll. (MZUSP 17675); (-9.6375, -65.0567), 5.MAR.2010, 106 m, TF Carrijo and RG Santos coll. (MZUSP 13213**). FRENCH GUIANA. Cayenne, Sinnamary, (5.0675, -53.0592), 4.FEB.2008, 34 m, J. Křeček coll. (UFTC FG277); Sinnamary, Petit Saut road, (5.10952, -52.96583), FEB.2019, 86 m, Y Roisin, C Legrand coll. (ULB Pb19-30B**); Régina, Camp Patawa, (4.541, -52.157), 11.FEB.2007, T Bourguignon coll. (ULB Qua3-1.3-3); Régina, Nouragues Inselberg Station, (4.0852, -52.6815), 15.JAN.2010, T Bourguignon, Y Roisin, J Šobotník, R. Hanus, J Cvačka coll. (ULB G451). PERU. Pasco, Kirishari, (-10.155210, -75.009120), 27.MAY.2014, 272 m, TF Carrijo, JA Chase, R Constantino, JR Mangold, A Mullins, J Křeček, S Kuswanto, T Nishimura, and RH Scheffrahn coll. (UFTC PU393).

##### Diagnosis.

The EVA has a “star-like” plaque attached to only one cushion; all cushions are striated. The cushions are adorned with a few small spines in the proximal portion (P1 junction). The mixed segment has an inflated mesenteric tongue.

##### Description.

**Figure 1.**
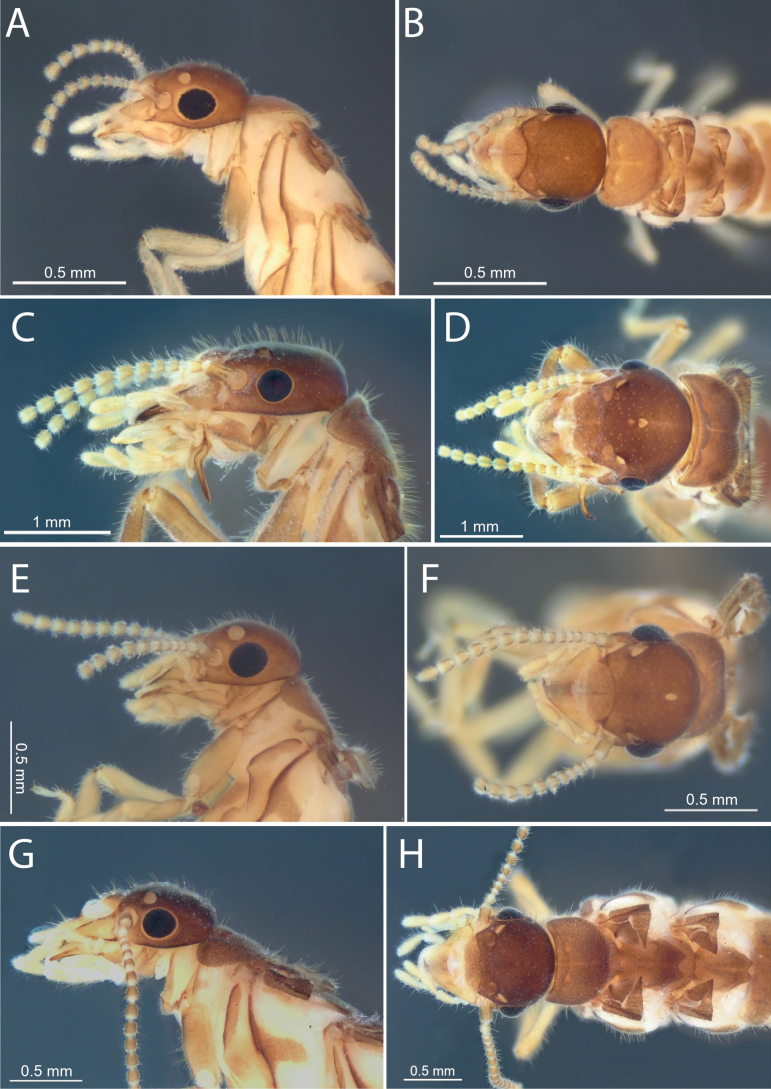
Imago head and pronotum in dorsal and lateral view **A, B***Anoplotermessusanae* sp. nov. **C, D***Hirsutitermeskanzakii* sp. nov. **E, F***Krecekitermesdaironi* sp. nov. **G, H***Ourissotermesgiblinorum* sp. nov.

***Imago*** (Fig. 1A, B, Table 1). Very small. Head capsule pale reddish, covered with numerous short hairs and a few sparse bristles. Eye elliptic, with the dorsoventral diameter shorter. Ocelli circular, large relative to eye. Fontanelle oval, very small. Postclypeus barely inflated, pale brown, concolorous with pro-, meso-, and metanotum. Anteclypeus narrow with anterolateral margins slightly concave, tip rounded. Antenna with 12 articles (formula 2>3≈4<5). Pronotum with short bristles along margins and very short hairs on surface; anterior margin straight, with median depression. Meso- and metanotum covered with numerous short hairs.

**Table 1.** Measurements of imagoes (range and mean in mm). L: length; W: width; c = number of colonies; *n* = number of individuals.

	*A.susanae* ♀ (c = 2, *n* = 3)	*A.susanae* ♂ (c = 1, *n* = 1)	*H.kanzakii* ♂ (c = 1, *n* = 1)	*K.daironi* ♀ (c = 1, *n* = 2)	*K.daironi* ♂ (c = 1, *n* = 1)	*O.giblinorum* ♀ (c = 1, *n* = 3)	*O.giblinorum* ♂ (c = 1, *n* = 2)
Head length to lateral base of mandibles	0.41–0.43 (0.44)	0.37	1.10	0.46–0.48 (0.47)	0.55	0.6–0.63 (0.61)	0.63–0.68 (0.65)
Maximum head width	0.42–0.44 (0.43)	0.42	1.42	0.50–0.54 (0.52)	0.55	0.78–0.8 (0.79)	0.73–0.75 (0.74)
Inter-eye distance	0.40–0.41 (0.40)	0.04	1.27	0.34–0.37 (0.36)	0.54	0.73–0.75 (0.74)	0.70–0.73 (0.71)
Max. diameter ocellus	0.07–0.08 (0.07)	0.07	0.15	0.07–0.08 (0.07)	0.12	0.13–0.13 (0.13)	0.10–0.11 (0.10)
Min. diameter ocellus	0.05–0.06 (0.06)	0.05	0.10	0.05–0.05 (0.05)	0.09	0.09–0.10 (0.10)	0.08–0.09 (0.08)
Max. diameter eye	0.19–0.20 (0.19)	0.17	0.37	0.19–0.22 (0.21)	0.25	0.25–0.28 (0.26)	0.26–0.28 (0.27)
Min. diameter eye	0.15–0.17 (0.16)	0.15	0.35	0.16–0.16 (0.16)	0.20	0.23–0.25 (0.24)	0.23–0.24 (0.23)
Pronotum length	0.27–0.30 (0.28)	0.27	0.77	0.34–0.35 (0.34)	0.37	0.48–0.50 (0.49)	0.45–0.48 (0.46)
Pronotum width	0.41–0.45 (0.43)	0.42	1.35	0.42–0.43 (0.42)	0.55	0.73–0.78 (0.76)	0.71–0.73 (0.72)
Hind tibia length	0.45–0.47 (0.47)	0.37	1.725	0.35–0.40 (0.37)	0.70	0.98–1.05 (1.01)	0.98–1.05 (1.01)
Fore tibia length	0.37–0.37 (0.37)	0.37	1.325	0.36–0.38 (0.37)	0.55	0.75–0.75 (0.75)	0.70–0.70 (0.70)
Fore tibia width	0.06–0.07 (0.07)	0.07	0.19	0.07–0.07 (0.07)	0.12	0.13–0.13 (0.13)	0.13–0.13 (0.13)
Fore tibia W/L ratio	0.17–0.20 (0.19)	0.20	0.14	0.19–0.19 (0.19)	0.23	0.173–0.173 (0.17)	0.185–0.185 (0.18)

**Figure 2.**
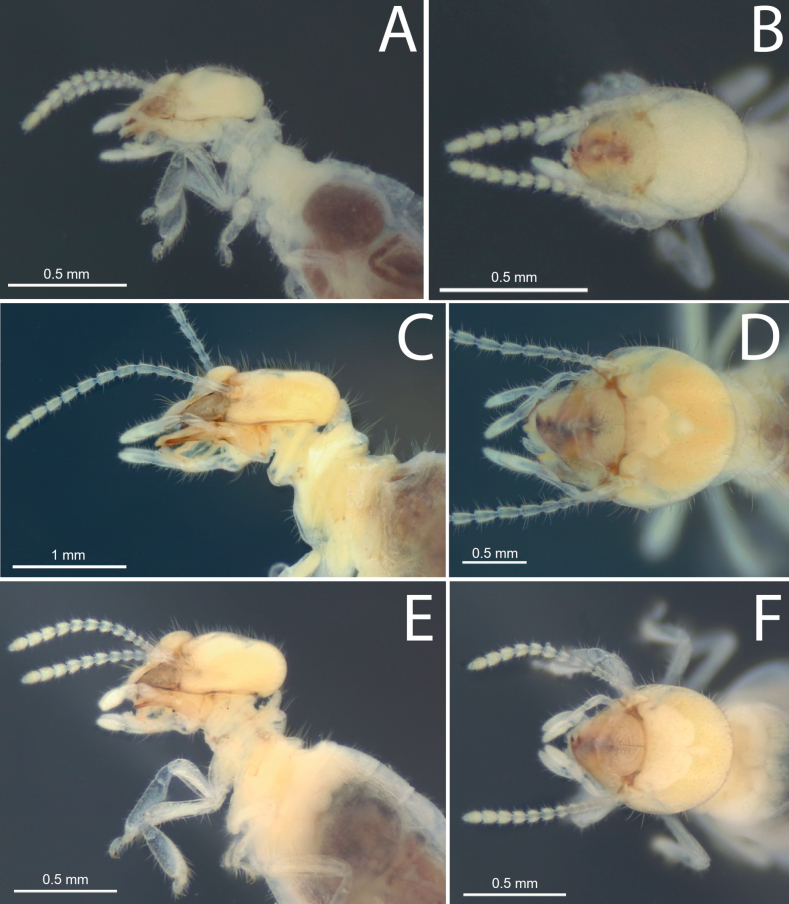
Head capsule of workers **A, B***Anoplotermessusanae* sp. nov. **C, D***Hirsutitermeskanzakii* sp. nov. **E, F***Krecekitermesdaironi* sp. nov.

***Worker*** (Fig. 2A, B, Table 2). Monomorphic. Very small. Head capsule and antennal articles whitish. Fontanelle inconspicuous. Antenna with 12 or 13 articles. Postclypeus moderately inflated. Head capsule covered with numerous short hairs and sparse longer setae on anteclypeus and postclypeus. Left mandible with apical tooth more prominent than (M1+2), gap between the two forming acute angle; posterior margin of M1+2 weakly concave; M3 (third marginal) less prominent than M1+2, edges forming an acute angle; molar prominence well developed, eclipsing molar process in dorsal view. Right mandible with apical tooth more prominent than M1; gap between M1 and M2 forming right angle, M2 with concave posterior margin (Fig. 5). Pronotum with long bristles and short hairs in the margins and surface. Tergites and sternites with short and microscopic hairs on the surface in the margins. Fore tibia (Fig. 4A) strongly inflated, short; inner surface with a line of long bristles from mid-length to apex.

**Table 2.** Measurements of workers (range and mean in mm). LH bm: length of head to lateral base of mandibles; WH max: maximum width of head; LHT: length of hind tibia; LPT: length of protibia; WPT: width of protibia; RPT: ratio WL of protibia. c = number of colonies; *n* = number of individuals.

Species	LH bm	WH max	LHT	LPT	WPT	RPT
*Anoplotermessusanae* (c = 3, *n* = 10)	0.26–0.41 (0.35)	0.43–0.60 (0.50)	0.30–0.46 (0.36)	0.29–0.40 (0.33)	0.09–0.14 (0.10)	0.22–0.38 (0.32)
*Hirsutitermeskanzakii* (c = 8, *n* = 21)	0.53–1.40 (0.83)	0.90–1.28 (1.13)	0.61–1.28 (0.91)	0.68–1.05 (0.85)	0.16–0.29 (0.22)	0.20–0.31 (0.25)
*Krecekitermesdaironi* (c = 10, *n* = 35)	0.26–0.55 (0.40)	0.57–0.63 (0.61)	0.32–0.47 (0.40)	0.35–0.42 (0.39)	0.10–0.12 (0.11)	0.24–0.33 (0.29)
*Mangolditermescurveileum* (c = 9, *n* = 56)	0.73–0.99 (0.82)	0.75–1.40 (1.13)	0.45–1.05 (0.76)	0.4–0.91 (0.68)	0.12–0.27 (0.21)	0.25–0.41 (0.32)
*Ourissotermesgiblinorum* (c = 3, *n* = 10)	0.58–0.70 (0.64)	0.70–0.80 (0.76)	0.42–0.80 (0.69)	0.48–0.65 (0.60)	0.17–0.23 (0.19)	0.28–0.37 (0.32)

Gut (Fig. 6A) elongated; mixed segment (MS): mesenteric tongue with a whitish spherical mesenteric nodule in ventral view. First proctodeal segment (P1) width uniform along its entire length. Enteric valve seating (EVS) elongated and cylindrical; armed with a “star-like” plaque on only one cushion; the plaque projects 16–20 spines along its margin and 1–3 surface spines. Cushions and inter-cushions vertically striated; proximal part of the cushion is smooth, without ornamentation (for this reason it gives the appearance of wrinkled fabric, which is very common); cushions with 5–7 short spines are in the distal part of the cushion directed backward, in the inflated central part of the cushion. Enteric valve seating filled with bacteria located on “bacterial slime”.

##### Comparison and remarks.

*Anoplotermessusanae* can be distinguished from all other *Anoplotermes* species by the “star-like” plaque of the EVA. Although *Anoplotermesparvus* also has a sclerotized plate on the EVA, it forms an asymmetrical spiny mass (Fig. 11G).

##### Molecular analysis.

The phylogeny recovered this species as the sister group of the four other *Anoplotermes* species (including the type species) included in this study, except *A.meridianus*, which was recovered as the sister group of *Humutermes*. The EVA and COI sequence alone suggested this species should be placed in a new genus; however, the external and gut morphology, and the complete mitogenome, corroborate it as a new *Anoplotermes* species.

##### Field observation and distribution.

This species is distributed in Ecuador, Brazil, and French Guiana (Fig. 13). It has been found mainly on the ground and in small epigeous nests (not confirmed if its own nest). On one occasion, *A.susanae* was found adjacent to workers of an *Anhangatermes* species. Other studies have reported this species as morphospecies under the name of *Anoplotermes* grp AC in Ecuador (Dahlsjö et al. 2020) and *Anoplotermes*-group sp. AK in French Guiana (Bourguignon et al. 2011a, 2013, 2015).

##### Etymology.

This species is named in honor of Susan G. Scheffrahn, wife of RHS.

#### ﻿ Hirsutitermes

Taxon classificationAnimaliaIsopteraTermitidae

Scheffrahn, Carrijo & Castro gen. nov.

63B7EBFC-04F4-5AD7-BB8E-09463FF03065

https://zoobank.org/AE425206-7F9D-43FB-B1B4-CBE022274F85

##### Type species.

*Hirsutitermeskanzakii* sp. nov.

##### Diagnosis.

Enteric valve armature (EVA) embellished with hundreds of thin spines on the posterior rim. The EVA seating is tubular, trilobed, and long. The mesenteric tongue is long but not inflated.

##### Description.

***Imago*** (Fig. 1C, D, Table 1). Head forms a very gentle curve in lateral view. Fontanelle conspicuous, heart-shaped, situated near a virtual line from the posterior margin of eyes, behind an oval-shaped spot (= labral medial muscle). Two conspicuous oval patches above antennal sockets. Antenna with 12 articles (formula 2>3<4≈5). Pronotum with median suture well marked; anterior margin raised.

***Worker*** (Fig. 2C, D, Table 2). Fontanelle rounded, conspicuous but with faint margins. Postclypeus moderately inflated. Antenna with 14 articles (formula 2>3=4<5). Left mandible with apical tooth more prominent than M1+2; margins of M3 forming an obtuse angle with the tip slightly rounded and directed forward; molar process conspicuous and not eclipsed by the molar prominence; molar prominence well developed. Right mandible with apical tooth much more prominent than M1; M1 small, less prominent than M2; M2 margins curved; molar plate concave, with a few ridges – not visible in the figure (Fig. 5B). Gut with long mesenteric tongue but not inflated; EVA seating tubular, trilobed, and long (Fig. 6B); EVA ornamented with hundreds of thin spines on the posterior portion of the cushions (Fig. 9A–C).

##### Comparison and remarks.

The EVA of *Hirsutitermes* is closest to that of *Longustitermes*; however, the spines of *Hirsutitermes* are longer and thinner than those of *Longustitermes* and the anterior portion of the cushions is more rounded in the latter than pyriform in the former. In addition, the external morphology of these two genera is very different, *Longustitermes* is smaller in size and has a trilobed EVS, while *Hirsutitermes* has a tubular EVS.

##### Molecular analysis.

The position of the genus is not clear. In the BI analysis, *Hirsutitermes* was recovered as the sister group of the clade composed of *Krecekitermes* and *Anoplotermes* with a posterior probability of 0.96 (Fig. 12). In the ML analysis, the genus was recovered as sister group to all New World Apicotermitinae (Suppl. material 2).

##### Etymology.

From the Latin *hirsutus*, meaning hairy, rough, refers to the hirsute appearance of the EVA armature.

#### ﻿ Hirsutitermes kanzakii

Taxon classificationAnimaliaIsopteraTermitidae

Scheffrahn, Carrijo & Castro sp. nov.

02BA0AEB-EB83-5768-8105-FF642FC7E57C

https://zoobank.org/E30B927B-F291-4931-8295-F1213838BF5E

##### Material examined.

***Holotype*.** Worker from lot UNAB 6137 (in a separate vial with the remaining sample).

##### Type locality.

Colombia. Caquetá, Florencia, Palmichar, 1.7145, -75.6148.

##### Type repository.

UNAB.

***Paratypes*.** Bolivia. Beni, Puerto Leigue, (-14.2126, -64.9402), 29.MAY.2013, 149 m, TF Carrijo, JA Chase, R Constantino, JR Mangold, A Mullins, J Křeček, T Nishimura, and RH Scheffrahn coll. (UFTC BO532). Brazil. Rondônia, Porto Velho, Abunã, (-9.6341, -65.4513), 11.MAR.2010, 123 m, TF Carrijo and RG Santos coll. (MZUSP 13054); (-9.632, -65.4387), 18.MAY.2010, 113 m, TF Carrijo and MM Rocha coll. (MZUSP 17968); (-9.6422, -65.4462), 28.JUN.2010, 104 m, TF Carrijo and SP Rosa coll. (MZUSP 17970**); (-9.6341, -65.4513), 29.JUN.2010, 123 m, TF Carrijo and SP Rosa coll. (MZUSP 17971); (-9.632, -65.4387), 5.APR.2011, 113 m, VTC Mercado and RS Probst coll. (MZUSP 17973); Porto Velho, Jaci Paraná, (-9.4502, -64.3674), 20.SEP.2010, TF Carrijo and RG Santos coll. (MZUSP 17976); (-9.4502, -64.3674), 12.JAN.2011, RG Santos and CY Mandai coll. (MZUSP 17977); Porto Velho, Nova Mutum-Paraná, (-9.3209, -64.7237), 18.SEP.2010, 96 m, TF Carrijo and RG Santos coll. (MZUSP 17975**). Colombia. Caquetá, Florencia, Palmichar, (1.7145, -75.6148), 23.MAR.2016, 241 m, Y Virguez coll. (CATAC-1801, UNAB 6137); Bajo Caldas, (1.6481, -75.6360), 12.DEC.2018, 429 m, M Perez coll. (CATAC-4673); Bélen de los Andaquies, Agua Dulce, (1.3372, -75.8094), 23.APR.2018, 262 m, J Murillo coll. (CATAC-6745). French Guiana. Cayenne, Régina, Nouragues Inselberg Station, (4.0904, -52.6772), 23.JAN.2010, T Bourguignon, Y Roisin, J Šobotník, R. Hanus, J Cvačka coll. (ULB G627); Sinnamary, Petit Saut road, (5.11056, -52.96540), FEB.2019, 72 m, Y Roisin, C Legrand, P Babzenko, N Kaczmarek coll. (ULB L05-10C**). Trinidad and Tobago. Tunapuna-Piarco, Blanchisseuse, (10.7963, -61.2826), 26.MAY.2003, 47 m, JA Chase, J Křeček, B Maharajh, JR Mangold, RH Scheffrahn, and J Warner coll. (UFTC TT1357**); Tunapuna, (10.6675, -61.3991), 10.JUN.2004, 315 m, J Davis coll. (UFTC TT2166). Venezuela. Sucre, San Esteban, (10.3013, -64.3758), 28.SEP.2007, JA Chase, JR Mangold, and RH Scheffrahn coll. (UFTC VZ363).

##### Diagnosis.

As described for the genus.

##### Description.

***Imago*** (Fig. 1C, D, Table 1). Medium size. Head capsule brown, covered with dozens of long bristles and microscopic hairs, bristle bases marked. Eyes sub-circular and relatively small. Ocelli sub-oval of medium-size and far away from the eyes. Postclypeus modestly inflated and concolorous with oval patches. Pronotum with the posterior margin emarginated, densely covered with long and short hairs on the margins and surface. Meso- and metanotum densely covered with numerous long hairs.

***Worker*** (Fig. 2C, D, Table 2). Monomorphic. Medium size. Body and head size range variable, from medium to large. Head capsule pale yellow, covered with numerous long hairs and sparse bristles of slightly larger size; dorsal surface flat in lateral view. Antennal articles translucent white. Pronotum with very long bristles, especially in the middle of anterior and posterior lobes. Tergites and sternites with short hairs on the surface and long bristles in the posterior margins. Forecoxa and fore femur with medium-sized spines. Fore tibia (Fig. 4B) moderately inflated and heavily covered with spines and setae of different sizes.

Gut (Fig. 6B) with long mesenteric tongue, not inflated. Enteric valve seating relatively long and tubular, often with three distinct but small lobes; P3-a/enteric valve seating (EVS) junction visible in dorsal view; EVA armed (Fig. 9A–C) with hundreds of long, slender, sclerotized spines arranged from the fourth portion near of the EVA distal portion up to tip of cushions and inter-cushion cuticle. Cushions pyriform pouches with about 20 spicules; scales between pouches and spines with microscopic fringes on posterior margins.

##### Remarks.

See remarks for the genus.

##### Ecology and distribution.

*Hirsutitermeskanzakii* is distributed from Bolivia to Trinidad and Tobago. The material examined for this study did not include samples from Ecuador, but Dahlsjö et al. (2020) reported this species as *Anoplotermes* grp OI. Likewise, previous studies reported this species as *Anoplotermes*-group sp AN in French Guiana (Bourguignon et al. 2011a, b, 2013, 2015) and Apicotermitinae sp. 2 in Colombia (Castro et al. 2021). This species has a wide distribution in greater Amazonia where it is found in the soil. In Colombia, it is rather common in natural forests but uncommon in young rubber plantations (Castro et al. 2021).

##### Etymology.

This species is named in honor of Dr. Natsumi Kanzaki, a Japanese nematologist who has a keen interest in the nematodes of termites.

#### ﻿ Krecekitermes

Taxon classificationAnimaliaIsopteraTermitidae

Scheffrahn, Carrijo & Castro gen. nov.

857B03C3-DF24-564C-A828-D0B9CC97A4BF

https://zoobank.org/9B87B114-5815-4547-82F2-D3509BFCCE30

##### Type species.

*Krecekitermesdaironi* sp. nov.

##### Diagnosis.

Dehiscent organs fill the hemocoel anterior to the crop. The EVS forms three large lobes. The Mesenteron/P1 junction is well marked, but the mixed segment is short. The EVA armature of *Krecekitermes* is composed of cushions with six sclerotized and crown-shaped termini adorned with thorns.

##### Description.

***Imago*** (Fig. 1E, F, Table 1). Dorsal surface of the head capsule concave in lateral view. Fontanelle very conspicuous. Postclypeus barely inflated, with the median suture well-marked. Antenna with 14 or 15 articles (formula 2>3<4≈5<6).

***Worker*** (Fig. 2E, F, Table 2). Head dorsal surface slightly concave in lateral view. Antenna whitish, with 13 or 14 articles, 2>3<4<5. Postclypeus robustly inflated. Left mandible with the apical tooth just as prominent as M1+2; edges of M3 forming an obtuse angle; truncated tip pointing backward; molar prominence well developed and prominent, hiding the molar process completely in dorsal view. Right mandible with apical tooth more prominent than M1; M1 well developed; M2 with prominent round tip pointing backward; margin between M1 and M2 forming an obtuse angle (Fig. 5C). Dehiscent organs conspicuous in most individuals. Gut (Fig. 6C) with short mixed segment, thickening in the mesenteric tongue. The EVA armature is composed of six sclerotized and crown-shaped termini adorned with thorns in the posterior part of the cushions.

##### Comparison and remarks.

*Dissimulitermes* and *Ruptitermes* present dehiscent organs like *Krecekitermes*; however, *Ruptitermes* does not present armature in the EVA, except for *R.bandeirai*, but this species is much larger, and the spines of the EVA armature are concentrated in only three cushions. *Dissimulitermes* presents an EVA armature with sclerotized plates and *Krecekitermes* an EVA armature formed only by spines, without plates. Enteric valve seating (EVS) is tubular in *Dissimulitermes* and is trilobed in *Krecekitermes*.

##### Molecular analysis.

This genus was recovered as the sister group of *Anoplotermes*, in a clade composed of *Hirsutitermes* + *Krecekitermes* + *Anoplotermes* (Fig. 12). But the position of *Hirsutitermes* was divergent between ML and BI analyses (Fig. 12, Suppl. material 2).

##### Etymology.

We named this genus in honor of Dr. Jan Křeček, a retired Czech terminologist who, along with RHS, contributed to many termite descriptions while at the University of Florida, Ft. Lauderdale R.E.C.

#### ﻿ Krecekitermes daironi

Taxon classificationAnimaliaIsopteraTermitidae

Scheffrahn, Carrijo & Castro sp. nov.

9AF682AF-135A-5583-957D-C302B1DDACFD

https://zoobank.org/7E2B2BA3-10C0-4092-AD88-495CF79E2493

##### Material examined.

***Holotype*.** Worker from lot MZUSP 17407** (in a separate vial with the remaining sample).

##### Type locality.

Brazil. Rondônia, Porto Velho, Mutum-Paraná, -9.4513, -64.8439.

##### Type repository.

MZUSP.

***Paratypes*.** Bolivia. Cochabamba, Chapare, Villa Tunari, (-16.9704, -65.2100), 26.MAY.2013, 247 m, TF Carrijo, JA Chase, R Constantino, JR Mangold, A Mullins, J Křeček, S. Kuswanto, T Nishimura, and RH Scheffrahn coll. (UFTC BO84); (-17.0024, -65.4356), 26.MAY.2013, 332 m, TF Carrijo, JA Chase, R Constantino, JR Mangold, A Mullins, J Křeček, S. Kuswanto, T Nishimura, and RH Scheffrahn coll. (UFTC BO116, BO119). Brazil. Rondônia, Porto Velho, Abunã, (-9.5965, -65.3371), 15.MAY.2010, 99 m, TF Carrijo and MM Rocha coll. (MZUSP 17377); (-9.6422, -65.4462), 10.MAR.2010, 103 m, TF Carrijo and RG Santos coll. (MZUSP 13061); Porto Velho, Jaci Paraná, (-9.1627, -64.6211), 14.SEP.2010, 97 m, TF Carrijo and RG Santos coll. (MZUSP 17411); (-9.1466, -64.6307), 14.SEP.2010, 104 m, TF Carrijo and RG Santos coll. (MZUSP 18812); 30.MAR.2011, 104 m, RG Santos and CY Mandai coll. (MZUSP 18893); Porto Velho, Mutum-Paraná, (-9.4401, -64.7863), 27.FEB.2010, 101 m, TF Carrijo and RG Santos coll. (MZUSP 13193); 10.SEP.2010, 101 m, MM Rocha and VTC Mercado coll. (MZUSP 17397); (-9.4513, -64.8439), 30.MAR.2010, 112 m, MM Rocha and RG Santos coll. (MZUSP 17407**, 17408); (-9.4428, -64.7946), 28.FEB.2010, 98 m, TF Carrijo and RG Santos coll. (MZUSP 13190); (-9.61, -65.0567), 4.MAR.2010, 102 m, TF Carrijo and RG Santos coll. (MZUSP 13214); (-9.6069, -65.0458), 10.JAN.2011, 102 m, MM Rocha and LP Prado coll. (MZUSP 17400); (-9.5858, -65.0536), 26.JUN.2010, 206 m, TF Carrijo and SP Rosa coll. (MZUSP 17483**); 1.JAN.2011, 206 m, VTC Mercado and RS Probst coll. (MZUSP 18128); (-9.5791, -65.0579), 3.APR.2011, 136 m, VTC Mercado and RS Probst coll. (MZUSP 17402, 17403). Colombia. Amazonas, Puerto Nariño, (-3.7675, -70.3492), 17.JUL.2018, 95 m, JA Chase coll. (CATAC-3393). Caquetá, Belén de los Andaquíes, El Chocho, (1.4240, -75.7786), 31.JAN.2019, 286 m, M Pérez coll. (CATAC-6432); Morelia, San Marcos, (1.3900, -75.6511), 14.FEB.2019, 246 m, M Pérez coll. (CATAC-6575, UNAB 6135); San Vicente del Caguán, Buenos Aires, (2.069, -74.9334), 17.APR.2018, 399 m, J Murillo coll. (CATAC-7462). Vaupés, Mitú, Mituseño, (1.2265, -70.1219), 26.MAR.2019, 195 m, JA Chase coll. Ecuador. Guayas, Guayaquil, Bosque Protector Cerro Blanco, (-2.1809, -80.0196), 16.DIC.2001, 55 m, J Křeček coll. (UFTC EC1.1); Orellana, Tuptini, Yasuni station area (-0.67177, -76.39793), 29–31.APR.2011, 223 m, JA Chase, J. Křeček, JR Mangold, TJ Myles, A. Mullins, T. Nishimura, R Setter, and RH Scheffrahn coll. (UFTC EC517**, EC1037). French Guiana. Cayenne, Sinnamary, (5.0675, -53.0592), 4.FEB.2008, 34 m, J. Křeček coll. (UFTC FG277.4, FG726); (5.0238, -53.0248), 6.FEB.2008, 52 m, J. Křeček coll. (UFTC FG78, FG79); (5.07610, -53.02168), 04.JUN.2016, 90 m, Y Roisin, JE Romero Arias, S Hellemans coll. (ULB G16-128**); Régina, Nouragues Inselberg Station, (4.0833, -52.6815), 16.JAN.2010, T Bourguignon, Y Roisin, J Šobotník, R. Hanus, J Cvačka coll. (ULB G484). Peru. Madre de Dios, Reserva Nacional Tambopata, (-13.1370, -69.6120), 9.SEP.2015, L Carnohan coll. (UFTC PU1114, PU1126).

##### Diagnosis.

As described for the genus.

##### Description.

***Imago*.** (Fig. 1E, F, Table 1). Head capsule reddish brown, covered with bristles and short hairs. Eyes and ocelli sub-circular. Ocelli large, about the diameter of antennal sockets. Fontanelle oval-shaped and situated near a virtual line from the posterior margin of eyes. Pronotum elongated, covered with long bristles grouped on the margins and short hairs on the surface; lateral margins rounded.

***Worker*** (Fig. 2E, F, Table 2). Monomorphic. Medium to large-size. Fontanelle indistinct, small, and oval. Head capsule, anteclypeus, and postclypeus pale yellow, densely covered with numerous bristles. Pronotum with long bristles on the anterior lobe and a few very long bristles on the posterior lobe. Meso- and metanotum with very long bristles on the posterior margins. Tergites and sternites densely covered with short hairs on the surface. Fore tibia (Fig. 4C) moderately inflated and covered with short hairs and long bristles.

Dehiscent organs present, visible in most individuals. Gut (Fig. 6C) with short mixed segment, P1 of uniform width along the entire length. Enteric valve seating (EVS) strongly trilobed, with two lobes clearly visible on the right side of the abdomen; armature with six mildly sclerotized and heterogeneous crown-shaped cushions, with thorns on the distal tip of the cushions, three cushions are slightly larger and are interspersed with the smaller ones. One or occasionally two cushions with 1–6 thorns and five or four cushions with 8–13 spines; cushions elongated and inflated with 20–30 large fringed polygons scales; cuticle between cushions with 12–20 scales similar to those of the cushions; smaller scales with 6–15 unsclerotized points grouped at the base of the cushions (Fig. 9D–F).

##### Remarks.

See remarks for the genus.

##### Ecology and distribution.

This species is distributed from Bolivia to French Guiana. *Krecekitermes* was found in Amazonian and Pacific Forest (Fig. 13). Previous studies have reported this species as a morphospecies: *Anoplotermes*-group sp JU in Ecuador (Dahlsjö et al. 2020), *Anoplotermes*-group sp C in French Guiana (Bourguignon et al. 2009, 2011a, b, 2013, 2015), and Apicotermitinae sp. 3 in Colombia (Castro et al. 2021). With the records of these studies and our field notes, we conclude that this is an abundant species. In Bourguignon et al. (2011a), this is the species with the fourth highest occurrence among Apicotermitinae, collected in all sampling sites. In Castro et al. (2021), this is one of the Apicotermitinae species with the highest occurrence in plantations. Besides, *Krecekitermes* is a species that is found in natural and secondary forests with high occurrence, being found in young and mature rubber plantations and in recovering soils. This species was usually collected from the soil underneath stones and other surface objects.

##### Etymology.

This species is named in honor of the late Dairon Cárdenas (1957–2022), a Colombian botanist, co-founder of the Colombian Amazon Herbarium COAH of the SINCHI institute, who greatly contributed to the knowledge of the Amazon plants.

#### ﻿ Mangolditermes

Taxon classificationAnimaliaIsopteraTermitidae

Scheffrahn, Carrijo & Castro gen. nov.

79C7374E-A16C-58FA-9EC0-84CE4EF07351

https://zoobank.org/7BB54A83-6328-413C-967D-C6D03F901AC3

##### Type species.

*Mangolditermescurveileum* sp. nov.

##### Diagnosis.

In the left view, P1 makes a curve dorsally. The enteric valve seating (EVS) is weakly trilobed. The EVA is unarmed and composed of six elongated and inflated cushions, with well-marked fringed pentagonal scales in the distal half of the cushions. On the proximal ends, each cushion has about 10–20 tiny triangulate spines.

##### Description.

***Imago*.** Unknown.

**Figure 3.**
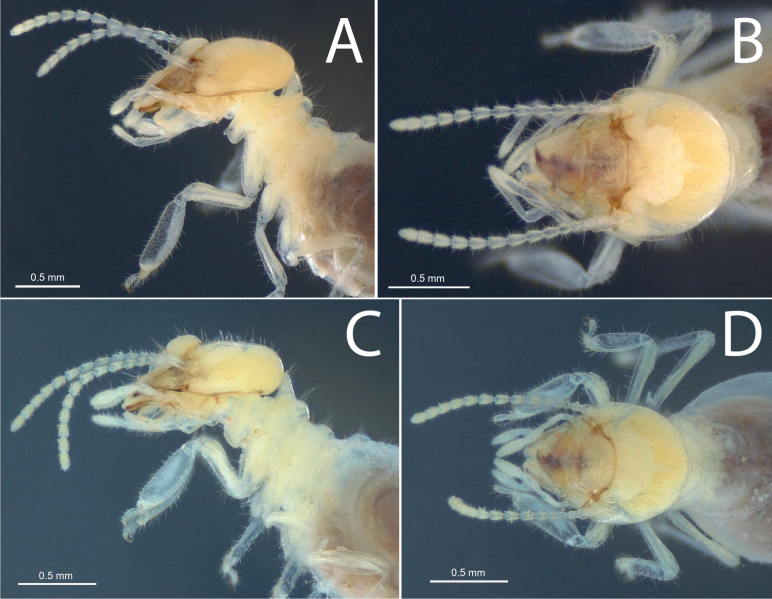
Head capsule of workers **A, B***Mangolditermescurveileum* sp. nov. **C, D***Ourissotermesgiblinorum* sp. nov.

***Worker*** (Fig. 3A, B, Table 2.) Fontanelle oval, with faint borders. Dorsal surface of head capsule slightly concave in lateral view. Antenna whitish, with 14 articles, 2<3≅4<5. Left mandible with apical tooth more prominent than M1+2; margin between M1+2 and apical tooth forming 75° angle; posterior margin of M1+2 nearly straight and long; molar process very acute, conspicuous dorsal view, and subequal to M3; molar prominence small. Right mandible with apical tooth much more prominent than M1; M1 with a very sharp point; M2 forming almost 90° angle, pointing backward; margin between M1 and M2 with a shallow incision, forming a wide slightly obtuse angle; molar plate reduced and concave (Fig. 5D). Gut (Fig. 7A) with small crop; mixed segment (MS) with a long, but not inflated, mesenteric tongue. In left view, P1 very long, S-shaped curve from the crop side to P3; enteric valve seating slightly trilobed. Enteric valve unarmed (Fig. 10A–C), composed of six elongated and inflated cushions, with well-marked fringed pentagonal scales in the distal half of the cushions.

##### Comparison and remarks.

*Mangolditermes* resembles *Aparatermes*, including the P1 shape, but can be distinguished by: 1) the mesenteric tongue (MT, mixed segment) morphology, that is elongated in the new genus and short in *Aparatermes*; 2) EVA with the pointed scales in the proximal portion of the cushions in *Mangolditermes* and towards the central portion of the cushion in *Aparatermes*; and 3) by the relatively inflated protibia of *Mangolditermes*. *Mangolditermes* has unarmed EVA, as *Hydrecotermes* and *Rustitermes*, but it can be separated from the latter two by its very long S-shaped P1 in left view. In ventral view, the mesenteric tongues of unarmed *Anoplotermes* have a whitish spherical mesenteric nodule, that is absent in *Mangolditermes*. Also, unarmed *Anoplotermes* have spatulate EVA cushions, while in *Mangolditermes* they are trapezoid and truncated at the base. *Disjunctitermes* can easily be confused with *Mangolditermes*; however, *Mangolditermes* is larger (WH max >0.75 mm) than *Disjunctitermes* (WH max < 0.7 mm); *Mangolditermes*EVA cushions are wider, while *Disjunctitermes* cushions are narrower, giving an elongated appearance. The cushions of the *Disjunctitermes* have very marked scales throughout the entire cushion, while in *Mangolditermes* the scales are only marked from the central to the anterior region of the cushion. *Tonsuritermes* is closely related to *Mangolditermes*, but *Tonsuritermes* workers have a very prominent fontanelle.

##### Molecular analysis.

This genus was recovered as the sister group of *Tonsuritermes*, with which it forms the sister group of a large clade composed of *Dissimulitermes*, *Disjunctitermes*, *Ourissotermes*, *Tetimatermes*, *Aparatermes*, *Compositermes*, and *Ruptitermesarboreus*. The last four form a well-corroborated group, but all the others should be interpreted as a polytomy.

##### Etymology.

We named this genus in honor of Dr. John Mangold, a retired American terminologist who collected and contributed to many Neotropical termite studies.

#### ﻿ Mangolditermes curveileum

Taxon classificationAnimaliaIsopteraTermitidae

Scheffrahn, Carrijo & Castro sp. nov.

74CC71F5-4CF7-5580-9A7A-300FC5884FA7

https://zoobank.org/B76ACDCF-C8F3-4970-A90C-4C3EC818084B

##### Material examined.

***Holotype*.** Worker from lot MZUSP 17079** (in a separate vial with the remaining sample).

##### Type locality.

Brazil. Rondônia, Porto Velho, Abunã, -9.6089, -65.3769.

##### Type repository.

MZUSP

***Paratypes*.** Bolivia. Santa Cruz, Ñuflo de Chaves, (-16.4935, -62.6529), 28.MAY.2013, 299 m, TF Carrijo, JA Chase, R Constantino, JR Mangold, A Mullins, J Křeček, T Nishimura, and RH Scheffrahn coll. (UFTC BO345). Brazil. Goiás, Arenópolis, (-16.3013, -51.4489), 21.JUL.2015, JP Constantini coll. (MZUSP 25623**); RG Santos coll. (MZUSP 23858**). Mato Grosso do Sul, Sonora, 23.JUL.2015, JP Constantini coll. (MZUSP 24047**). Rondônia, Porto Velho, (-9.0245, -64.2531), 7.JAN.2011, 84 m, RG Santos and CY Mandai coll. (MZUSP 17095, 17096); Porto Velho, Abunã, (-9.6321, -65.4387), 18.MAY.2010, 99 m, TF Carrijo and MM Rocha coll. (MZUSP 17101); (-9.6422, -65.4463), 28.JUN.2010, 105 m, TF Carrijo and SP Rosa coll. (MZUSP 17105, 17106); 10.MAR.2010, 105 m, TF Carrijo and RG Santos coll. (MZUSP 13043); (-9.6089, -65.3769), 11.JAN.2012, 103 m, RG Santos and JP Constantini coll. (MZUSP 17079**); (-9.6341, -65.4513), 11.MAR.2010, 123 m, TF Carrijo and RG Santos coll. (MZUSP 13047); Porto Velho, Jaci Paraná, (-9.1519, -64.5019), 15.JAN.2011, 89 m, RG Santos and CY Mandai coll. (MZUSP 17093, 17094); Porto Velho, Mutum-Paraná, (-9.5791, -65.0579), 3.APR.2011, 136 m, VTC Mercado and RS Probst coll. (MZUSP 17114, 17115); (-9.5824, -65.0687), 24.JUN.2012, 250 m, RG Santos and K Kawamishi coll. (MZUSP 17090). Roraima, Amajari, (3.6493, -61.7055), 13.MAR.2016, JP Constantini coll. (MZUSP 25247**). Colombia. Caquetá, Belén de los Andaquíes, (1.6278, -75.9047), 28.JAN.2017, 875 m, D Castro coll. (CATAC-0933, UNAB 6136); El Porvenir, (1.4758, -75.8677), 27.NOV.2018, 513 m, M Pérez coll. (CATAC-4164, CATAC-4170); Florencia, Bajo Caldas, (1.6482, -75.6361), 12.DEC.2018, 428 m, M Pérez coll. (CATAC-4700); Vaupés, Mitú, (1.2236, -70.1569), 28.MAR.2019, 183 m, CP Peña coll. (CATAC-9640). French Guiana. Cayenne, Sinnamary, Petit Saut road (5.0676, -52.9798), 13–18.FEB.2008, T Bourguignon coll. (ULB Qua5-4.3a); Saint-Laurent-du-Maroni, Saül, Monts la Fumée (3.6369, -53.2042), 30.MAY.2018, 260 m, Y Roisin, S Hellemans, N Kaczmarek coll. (ULB G18-178). Paraguay. Amambay, San Vicente, (-22.7078, -56.2882), 28–29.MAY.2012, 219 m, JA Chase, R Hickman, J Křeček, JR Mangold, A. Mullins, and RH Scheffrahn coll. (UFTC PA308, PA.309, PA312.1); (-22.6836, -56.2147), 29.MAY.2012, 225 m, JA Chase, R Hickman, J Křeček, JR Mangold, A. Mullins, and RH Scheffrahn coll. (UFTC PA393); Caaguazú, San José de los Arroyos, (-25.5085, -56.7896), 27.MAY.2012, 124 m, JA Chase, R Hickman, J Křeček, JR Mangold, A. Mullins, and RH Scheffrahn coll. (UFTC PA106); Cordillera, Ruta Nueva Colombia-Loma Grande, (-25.1747, -57.2876), 5.JUN.2012, 114 m, JA Chase, R Hickman, J Křeček, JR Mangold, A. Mullins, and RH Scheffrahn coll. (UFTC PA1268). Perú. Ucayali, Coronel Portillo, Campoverde, (-8.6085, -74.9362), 28.MAY.14, 186 m, T Carrijo, JA Chase, R Constantino, J Křeček, E Kuswanto, JR Mangold, A. Mullins, T. Nishimura, and RH Scheffrahn coll. (UFTC PU515, PU520); (-8.4886, -74.8584), 29.MAY.2014, 214 m, T Carrijo, JA Chase, R Constantino, J Křeček, E Kuswanto, JR Mangold, A. Mullins, T. Nishimura, and RH Scheffrahn coll. (UFTC PU667, PU668); (-8.5018, -74.8462), 29.MAY.2014, 213 m, T Carrijo, JA Chase, R Constantino, J Křeček, E Kuswanto, JR Mangold, A. Mullins, T. Nishimura, and RH Scheffrahn coll. (UFTC PU695, PU701, PU703, PU709.1); Nueva Requena, (-8.3700, -74.8436), 29.MAY.14, 185 m, T Carrijo, JA Chase, R Constantino, J Křeček, E Kuswanto, JR Mangold, A. Mullins, T. Nishimura, and RH Scheffrahn coll. (UFTC PU595, PU596). Trinidad and Tobago. Tunapuna-Piarco, Blanchisseuse, (10.7963, -61.2826), 26.MAY.2003, 45 m, JA Chase, J Křeček, B Maharajh, JR Mangold, RH Scheffrahn, and J Warner coll. (UFTC TT1324**); Sangre Grande, Mount Harris Forest, (10.4861, -61.1278), 31.MAY.2003, 114 m, JA Chase, J Křeček, B Maharajh, JR Mangold, RH Scheffrahn, and J Warner coll. (UFTC TT2080, TT2089).

##### Diagnosis.

As described for the genus.

##### Description.

***Imago*.** Unknown.

**Figure 4.**
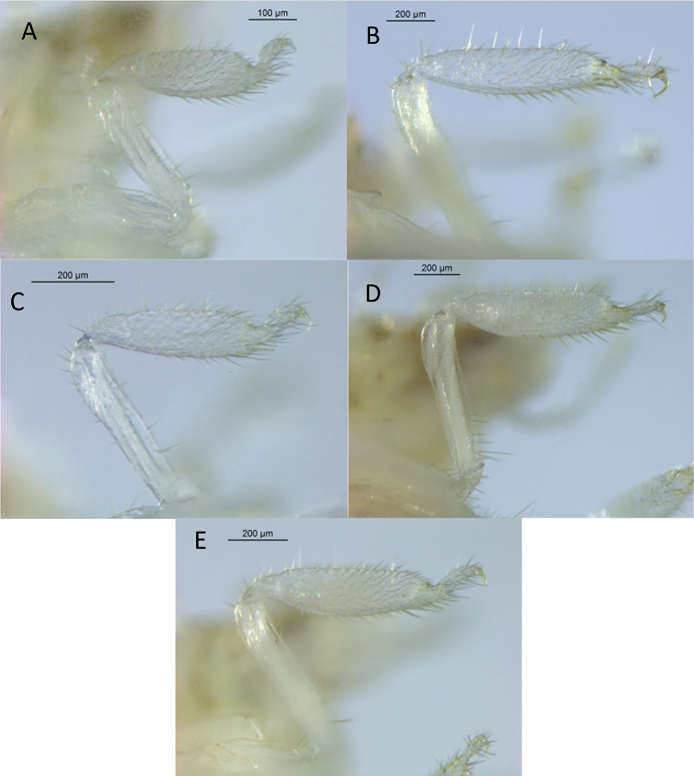
Foreleg of workers **A***Anoplotermessusanae* sp. nov. **B***Hirsutitermeskanzakii* sp. nov. **C***Krecekitermesdaironi* sp. nov. **D***Mangolditermescurveileum* sp. nov. **E***Ourissotermesgiblinorum* sp. nov.

***Worker*** (Fig. 3A, B, Table 2.) Monomorphic. Medium to large-size. Head capsule pale yellowish. Head, anteclypeus, and postclypeus covered with numerous bristles of variable size. Postclypeus moderately inflated. Pronotum with short stiff bristles on anterior margin; surface covered with long bristles. Posterior margins of pro-, meso-, and metanotum with long bristles. Fore femur with a few medium-sized bristles. Fore tibia (Fig. 4D) moderately inflated and densely covered with numerous longer and shorter bristles.

Gut (Fig. 7A) with mixed segment (MS) in left view, P1 very long, S-shaped curve from crop side to P3; EVAS slightly trilobed. Enteric valve unarmed (Fig. 10A–C), consisting of six inflated trapezoidal cushions; EVA with 18–25 well-marked cuboidal and fringed scales arranged in rows composed of 3–5 scales each; scales visible only in the distal half of the cushion; each cushion has about 10–20 tiny triangulate spines on the proximal end. Cuticle between the cushions without sculpture.

##### Remarks.

See remarks for the genus.

##### Ecology and distribution.

This species is widely distributed in South America and the Caribbean region. It is present from Paraguay to Trinidad and Tobago. It is also recorded from the Amazonian, Cerrado, and Chaco forests (Paraguay) (Fig. 13). This species was recorded as *Anoplotermes*-group sp AR by Bourguignon et al. (2011a). Collected mainly in the ground and foraging on highly decomposed pieces of wood.

##### Etymology.

The epithet *curveileum* is a compound noun formed from the words curved and ileum in reference to the curved P1 (the ileum), visible in the left view of the digestive tract.

#### ﻿ Ourissotermes

Taxon classificationAnimaliaIsopteraTermitidae

Scheffrahn, Carrijo & Castro gen. nov.

FD65614A-A29E-54D7-8E52-711D6248D5BB

https://zoobank.org/0502EECC-FF32-4CF5-B75C-6E1FE4361D65

##### Type species.

*Ourissotermesgiblinorum* sp. nov.

##### Diagnosis.

Enteric valve armature composed of six pectinate bunches with 4–6 rows of spines, distal portion (proximal to P3) with longer spines, decreasing posteriorly; smaller spines tooth-like. Proximal portion of cushions with a small spine tooth-like (near P1). Enteric valve (EV) inserted directly into the paunch (P3), without an enteric valve seating (EVS) differentiated.

##### Description.

***Imago*.** (Fig. 1G, H) Head slightly concave upwards the ocelli. Fontanelle very small or inconspicuous. Eye and ocelli subcircular. Antenna with 15 articles (formula 2>3<4<5<6). Lateral margin of pronotum elongated and well rounded.

**Figure 5.**
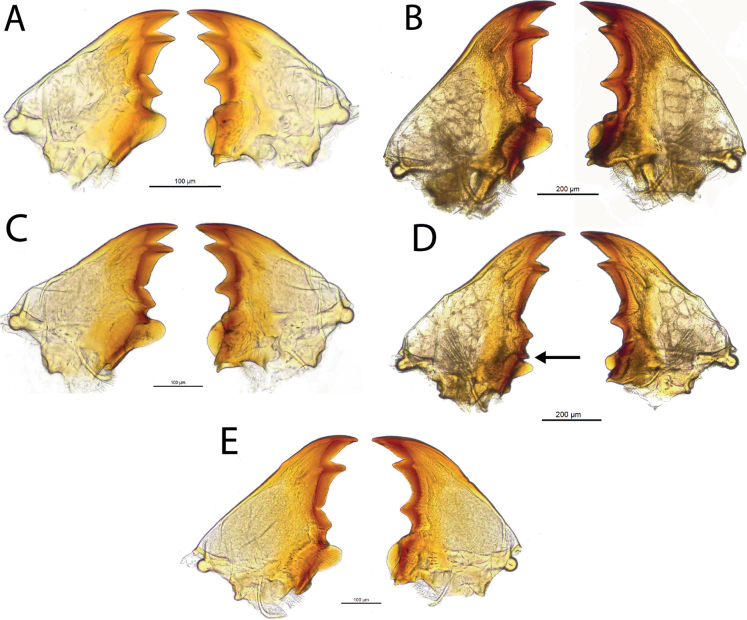
Mandibles of workers **A***Anoplotermessusanae* sp. nov. B *Hirsutitermeskanzakii* sp. nov. **C***Krecekitermesdaironi* sp. nov. **D***Mangolditermescurveileum* sp. nov. **E**, *Ourissotermesgiblinorum* sp.nov. Arrow indicates the molar process.

***Worker*.** (Fig. 3C, D) Dorsal surface of the head capsule slightly convex anteriorly in lateral view. Postclypeus highly inflated. Antenna with 14 articles. Left mandible with apical tooth more prominent than M1+2; edges of M3 forming a triangle with an obtuse angle and a slightly truncated tip; molar prominence well developed, partially hiding the molar process; right mandible with apical tooth more prominent than M1; M2 triangular, forming an acute angle with a prominent tip, but slightly less prominent than M1 (Fig. 5E). Gut (Fig. 7B) with a small mesenteric tongue and a simple transverse junction between P1 and the mesenteron; EVS not conspicuous. EVA composed of six pectinate bunches with 4–6 rows of spines, distal portion (proximal to P3) with longer spines, decreasing posteriorly.

**Figure 6.**
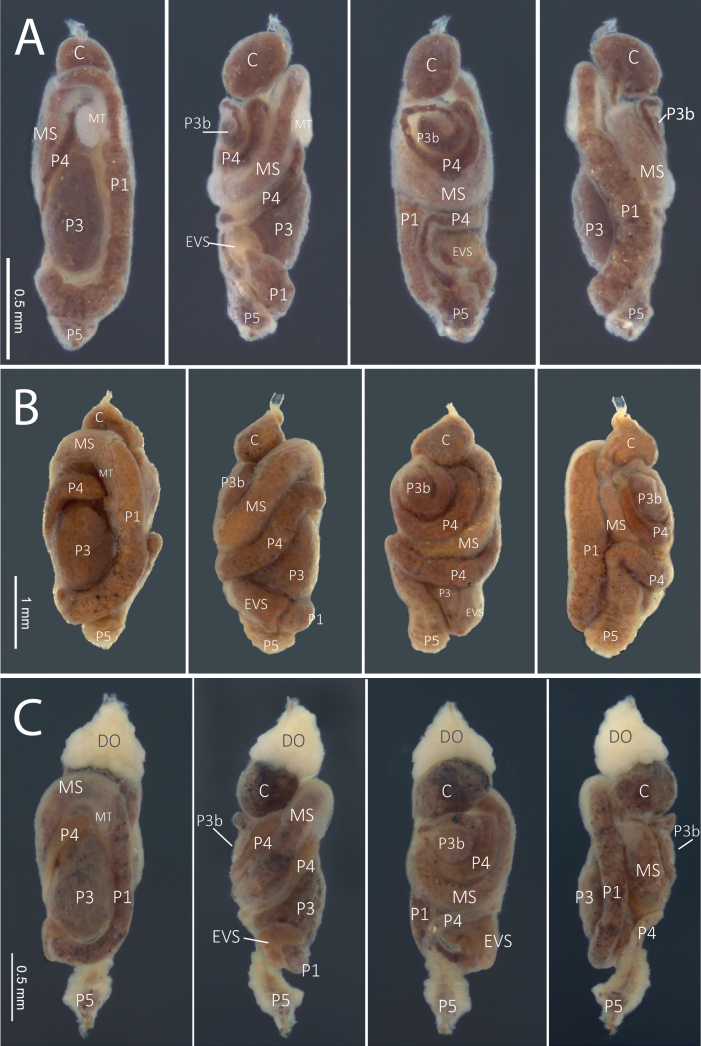
Worker digestive tracts. From left to right: ventral, right, dorsal, and left views **A***Anoplotermessusanae* sp. nov. **B***Hirsutitermeskanzakii* sp. nov. **C***Krecekitermesdaironi* sp. nov. Abbreviations: C = crop, DO = Dehiscent organ, MS = mesenteron, MT = mesenteric tongue (mixed segment), P1 = ileum, EVS = enteric valve seating, P3 and P3b = paunch, P4 = colon, P5 = rectum.

**Figure 7.** Worker digestive tracts **A***Mangolditermescurveileum* sp. nov. **B***Ourissotermesgiblinorum* sp. nov. From left to right: ventral, right, dorsal, and left views. Abbreviations: DO = dehiscent organ, C = crop, MS = mesenteron, MT = mesenteric tongue (mixed segment), P1 = ileum, EVS = enteric valve seating, P3 and P3b = paunch, P4 = colon, P5 = rectum.

##### Comparison and remarks.

Enteric valve armature of *Ourissotermes* is similar to *Patawatermes*, and *Echinotermes*EVA, but *Ourissotermes* can be easily differentiated because EVA is inserted directly in P3, without an EVS extension, while *Patawatermes*EVS is a tubular extension clearly visible externally; *Echinotermes* present a trilobed EVS. *Ourissotermes* present a digestive tract external morphology similar to that of *Hydrecotermes*; however, the EVA of *Hydrecotermes* is unarmed.

##### Molecular analysis.

This genus was recovered as part of a big clade composed of *Dissimulitermes*, *Disjunctitermes*, *Tonsuritermes*, *Mangolditermes*, *Tetimatermes*, *Aparatermes*, *Compositermes*, and the species *Ruptitermesarboreus*. The last four form a well-corroborated group, but all the others should be interpreted as a polytomy.

##### Etymology.

The species name is due to the resemblance of the EV armature with the body spines of a sea ​​urchin, that the translation to Portuguese is “ouriço”.

#### ﻿ Ourissotermes giblinorum

Taxon classificationAnimaliaIsopteraTermitidae

Scheffrahn, Carrijo & Castro sp. nov.

EBB0FD96-F87A-55BB-9351-3E7713C940F0

https://zoobank.org/858443DF-7180-4811-A25A-8C4F594EDBE7

##### Material examined.

***Holotype*.** Worker from lot MZUSP 17967 (in a separate vial with the remaining sample).

##### Type locality.

Brazil. Rondônia, Porto Velho, Jaci-Paraná, -9.4526, -64.3900.

##### Type repository.

MZUSP

***Paratypes*.** Bolivia. Cochabamba, Chapare, Villa Tunari, (-17.0024, -65.4356), 26.MAY.2013, 332 m, TF Carrijo, JA Chase, R Constantino, JR Mangold, A Mullins, J Křeček, T Nishimura, and RH Scheffrahn coll. (UFTC BO118, BO127, BO153); Cristal Mayu, (-16.9993, -65.6273), 26.MAY.2013, 504 m, TF Carrijo, JA Chase, R Constantino, JR Mangold, A Mullins, J Křeček, T Nishimura, and RH Scheffrahn coll. (UFTC BO167). Brazil. Goiás, Nazário, (-16.5904, -50.2291), 27.JUL.2015, TF Carrijo coll. (MZUSP 25404**); Minas Gerais, Presidente Olegário, (-18.3143, -46.5273), 18.JUL.2015, TF Carrijo coll. (MZUSP 25391**); Paraíba, João Pessoa, Mata do Buraquinho, (-7.1480, -34.8614), 01–20.JUN.2000, 63 m, A Vasconcellos coll. (MZUSP 13496); Rondônia, Porto Velho, Jaci Paraná, (-9.4526, -64.3900), 25.NOV.2011, 122 m, MM Rocha and J Cabral coll. (MZUSP 17967); Porto Velho, Nova Mutum Paraná, (-9.3176, -64.7269), 18.SEP.2010, 188 m, TF Carrijo and RG Santos coll. (MZUSP 17952**); Porto Velho, Mutum-Paraná, (-9.5823, -65.0686), 13.MAY.2010, 250 m, TF Carrijo and MM Rocha coll. (MZUSP 17949). Colombia. Vaupés, Mitú, Comunidad de Piracemo, (1.3375, -70.3889), 27.MAR.2019, 181 m, CP Peña coll. (CATAC-9447). FRENCH GUIANA. Cayenne, Sinnamary, Petit Saut road, (5.07388, -52.97933), 03.JUN.2016, 94 m, Y Roisin, JE Romero Arias, S Hellemans coll. (ULB G16-127); (5.11085, -52.96535), FEB.2019, 78 m, Y Roisin, C Legrand, P Babczenko, N Kaczmarek coll. (ULB L02-10F**); Régina, Nouragues Inselberg Station, (4.0833, -52.6815), 16.JAN.2010, T Bourguignon, Y Roisin, J Šobotník, R. Hanus, J Cvačka coll. (ULB G627). PERU, Ucayali, Nueva Requena, (-8.3700, -74.8436), 29.MAY.2014, 185 m, T Carrijo, JA Chase, R Constantino, J Křeček, E Kuswanto, JR Mangold, A. Mullins, T. Nishimura, and RH Scheffrahn coll. (UFTC PU611).

##### Diagnosis.

As described for the genus.

##### Description.

***Imago*.** (Fig. 1G, H) Head capsule, nota, and wing scales reddish-brown, with the head slightly darker. Head capsule with many bristles, densely covered by short hairs. Postclypeus slightly swollen, with a thin median suture. Anteclypeus elongated and wide, with rounded sides and medium portion. Pronotum with few short bristles in the margins. Meso- and metathorax covered with numerous hairs.

***Worker*.** (Fig. 3C, D) Medium-size. Monomorphic. Head capsule pale yellowish, with a faint fontanelle; antennal articles whitish. Head capsule covered with numerous medium-size bristles and sparse long setae. Pronotum with long bristles in the margins. Tergites and sternites with short hairs on the surface and long bristles in the margins. Fore tibia (Fig. 4E) moderately inflated and thickly covered with bristles of the same size, with sparse long-size bristles; pilosity denser apically with thicker spine-like bristles close to the tarsal region.

Gut (Fig. 7B) with a P1 of uniform width along the entire length, inserted directly into P3, making EVS not conspicuous. Each of six EV cushions armed with complex distal rake of sclerotized spines; rakes composed of 4–6 rows of pectinate spines, the posterior rows with 8–15 spines, and the anterior rows with 1–5 spines. Cushions pyriform, each with pentagonal and hexagonal scales; scales near the middle with small spine directed upwards; spines more noticeable near the distal portion; each cushion with a small spine directed proximally (Fig. 10D–F).

**Figure 8.**
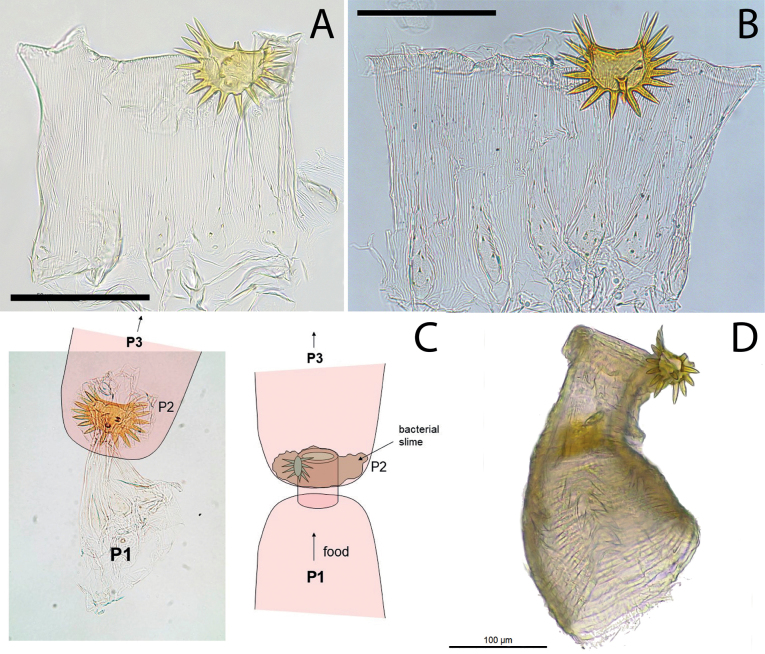
Worker enteric valve of *Anoplotermessusanae* sp. nov. **A, B** sliced mount **C** enteric valve seating filled with bacteria located on “bacterial slime”. Locations of enteric valve cushions (P2) and “star-like” plaque at the junction of P1 and P3 are diagrammed **D** whole mount. Scale bars: 100 μm.

**Figure 9.**
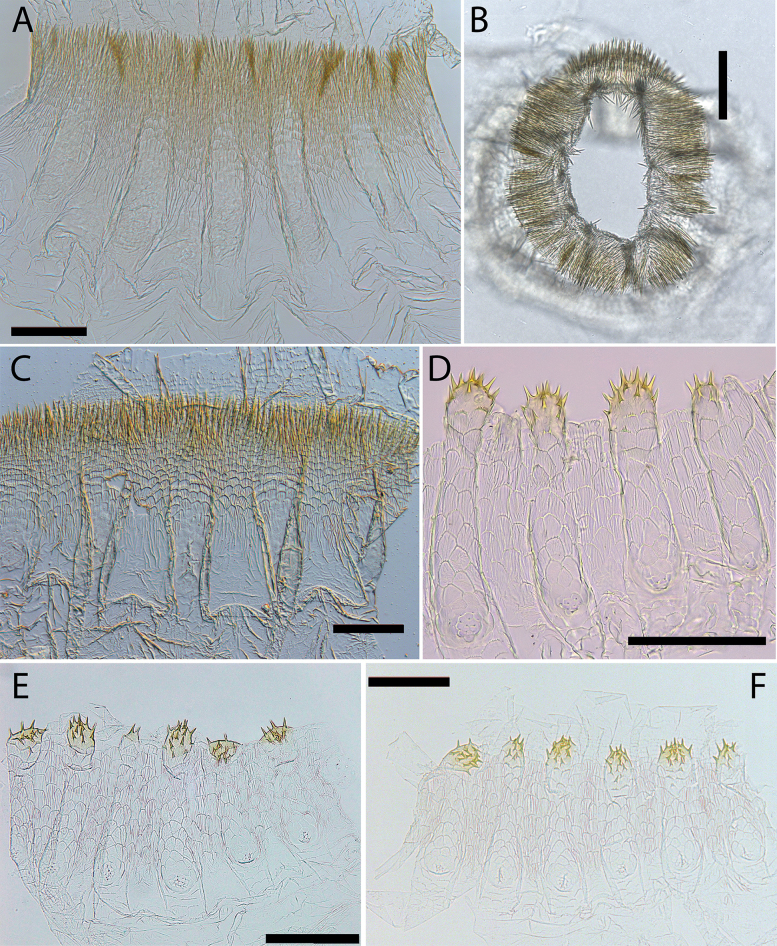
Worker enteric valves **A–C***Hirsutitermeskanzakii* sp. nov. from Colombia (UNAB 6137), Venezuela (UFTC VZ363), and Bolivia (UFTC BO532) **D–F***Krecekitermesdaironi* sp. nov. from Brazil (MZUSP 17402), Colombia (CATAC 8308), and Bolivia (UFTC BO84). Scale bars: 100 μm.

**Figure 10.**
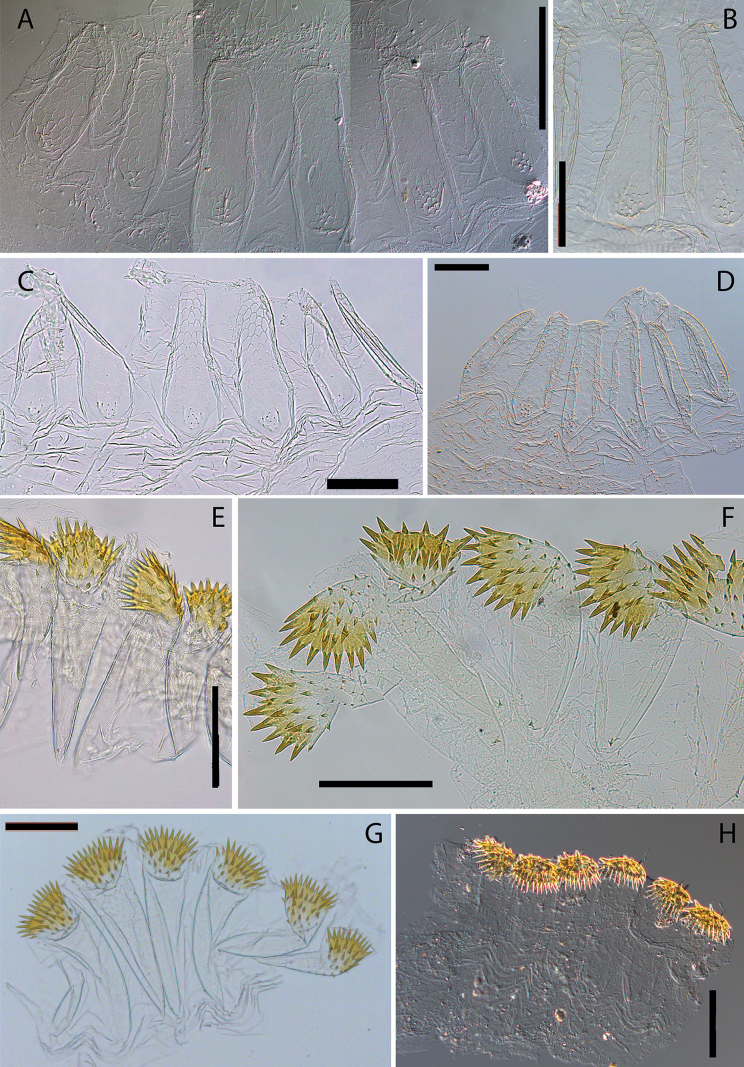
Worker enteric valves **A–D***Mangolditermescurveileum* sp. nov. from French Guiana (G18-178), Peru (UFTC PU520 and PU703), and Brazil (MZUSP 17114) **E–H***Ourissotermesgiblinorum* sp. nov. from Brazil (MZUSP 17967), Colombia (CATAC 9447), Bolivia (UFTC BO167), and French Guiana (G16-127). Scale bars: 100 μm.

**Figure 11.**
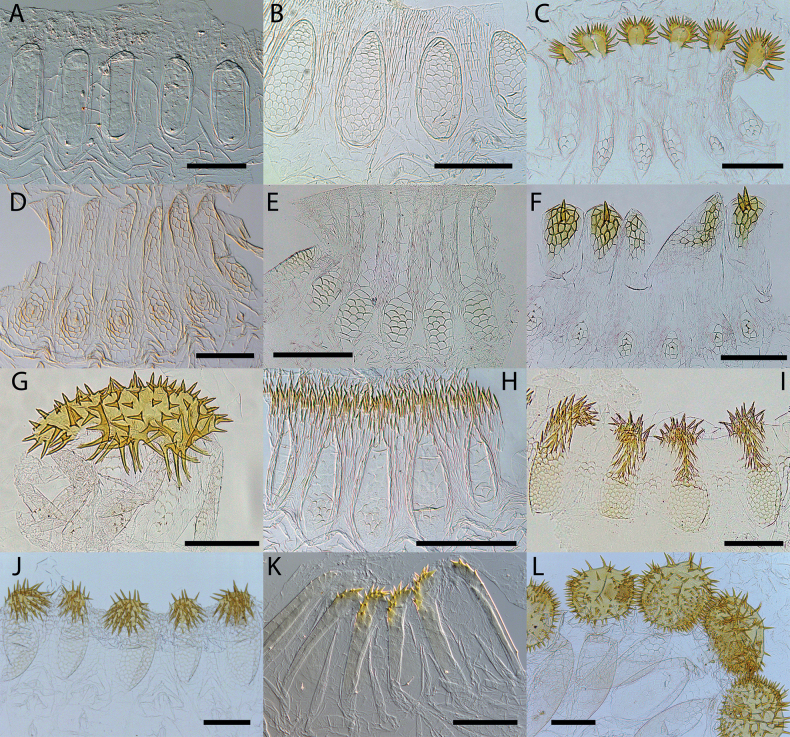
Apicotermitinae worker enteric valves **A***Hydrecotermesarienesho***B***Hydrecotermeskawaii***C***Humutermesnoiroti***D***Anoplotermesbanksi***E***Anoplotermesmeridianus***F***Anoplotermesjanus***G***Anoplotermesparvus***H***Longustitermesmanni***I***Patawatermesnigripunctatus***J***Patawatermesturricola***K***Rubeotermesjheringi***L***Grigiotermeshageni.* Scale bars: 100 μm.

##### Remarks.

See remarks for the genus.

##### Ecology and distribution.

This species was mainly collected from soil and decomposing wood pieces. It was found mainly in Amazon and Atlantic primary forests with a high state of conservation, at sites below 504 m a.s.l. This species was recorded as *Anoplotermes*-group sp. T in Bourguignon et al. (2009, 2011a, b, 2013, 2015).

##### Etymology.

This species is named in honor of Dr. Robin M. Giblin-Davis and his son Sean Giblin. From 1985 to 2020, “Rob” was a former colleague of RHS at the University of Florida, Ft. Lauderdale R.E.C., until his retirement. He taught RHS to appreciate nematodes in termites. For many years, Sean cleaned debris from thousands of termite vials upon the return of collection expeditions by RHS and colleagues listed in the “materials examined” section herein for UFTC samples. A complete listing of expeditions is available at Scheffrahn (2019).

### ﻿Key to neotropical Apicotermitinae genera based on the worker caste

Be aware before using this key: Many soldierless termite genera remain undescribed and are not included in this key. So, if your sample does not fit in any genus here, do not try to force a name to it. Using the EVA to confirm the identification is always recommended.

**Table:** 

1	Fontanelle large, sunken, diameter one quarter to three quarters head width. [see Constantini et al. (2018)]	***Tonsuritermes***
–	Fontanelle smaller, diameter less than one quarter head width. (Figs 2B, D, F, 3B, D)	**2**
2	Fore tibia with long “scooped out” excavation [see Fontes (1986)]	***Tetimatermes***
–	Fore tibia without long “scooped out” excavation	**3**
3	Enteric valve inserted directly in P3 (EVS not conspicuous in intact gut), P3 without tubular extension to P1 (Fig. 7B)	**4**
–	Enteric valve seating visible in intact gut (Figs 6A–C, 7A)	**5**
4	Enteric valve armed (Fig. 10D–F)	***Ourissotermes* gen. nov.**
–	Enteric valve unarmed (Fig. 11A, B)	***Hydrecotermes***
5	Junction of EVS/P3 with ring of 15–20 pectinate paddles [see Scheffrahn (2013), and (Carrijo et al. 2015)]	***Compositermes***
–	Junction of EVS/P3 without ring of 15–20 pectinate paddles	**6**
6	Mesenteric tongue (MS/P1 junction) with a whitish spherical mesenteric nodule in ventral view (Fig. 6A)	**7**
–	Mesenteric tongue without a nodule in ventral view (Figs 6B, C, 7A, B)	**8**
7	Enteric valve armature consists of six palmate pads (Fig. 11C)	***Humutermes***
–	Enteric valve armature with one to three sclerotized pads or enteric valve unarmed (Figs 8, 11D–G)	***Anoplotermes***
8	Dehiscent organs present (Fig. 6C)	**9**
–	Dehiscent organs absent (Fig. 6A, B)	**11**
9	Enteric valve unarmed; workers head pale yellow to black [See Acioli and Constantino (2015)]	***Ruptitermes*** ^***^
–	Enteric valve armed; workers head whitish to pale yellow	**10**
10	Enteric valve seating tubular, EVA armature pads with more than 20 spines [see Constantini et al. (2020)]	***Dissimulitermes***
–	Enteric valve seating trilobed (Fig. 6C), EVA armature pads with less than 13 spines (Fig. 8D–F)	***Krecekitermes* gen. nov.**
11	Enteric valve unarmed	**12**
–	Enteric valve armed	**15**
12	Mesenteric tongue spheroidal or elongated (Fig. 7A)	**13**
–	Mesenteric tongue short (drop shaped), not spheroidal (Fig. 6B, C)	**14**
13	Cushion scales are only marked from the central to the anterior portion of the cushion (Fig. 10A–C); larger, max head width > 0.7 mm	***Mangolditermes* gen. nov.**
–	Cushion scales markedly throughout the entire cushion [see Scheffrahn et al. (2017)]; smaller WH max < 0.7 mm	***Disjunctitermes***
14	EVA Cuticle between cushions with scales; EVS not trilobed [see Castro et al. (2020)]	***Rustitermes***
–	EVA Cuticle between cushions without scales; EVS trilobed [see Pinzón et al. (2019)]	***Aparatermes***
15	EVA armature resembling a continuous (spines on cuticle between cushions) wreath of hundreds of tiny and thin spines (Figs 9A–C, 11H)	**16**
–	EVA armature in form of comb, pads, or sclerotized plates on EVA cushions, not continuous on cuticle between cushions	**17**
16	Enteric valve seating tubular (Fig. 6B)	***Hirsutitermes* gen. nov.**
–	Enteric valve seating trilobed (Fig. 6C; see Bourguignon et al. 2010)	***Longustitermes***
17	EVA armature sclerotized plates, pads or comb present only in five cushions (Fig. 11K)	***Rubeotermes***
–	EVAA sclerotized plates, pads or comb present in all the six cushions	**18**
18	Enteric valve seating tubular (Fig. 6B)	**19**
–	Enteric valve seating trilobed (Fig. 6C)	**20**
19	Distal end of EVA armature pads capped with even tuft of spines; EVS elongated [Fig. 11I, J, see Bourguignon et al. (2016a)]	***Patawatermes***
–	Distal end of EVA armature pads ringed by unevenly arranged spines; EVS short [Fig. 11L, see Bourguignon et al. (2016a)]	***Grigiotermes***
20	EVA armature pads spiny, spherical [see Castro et al. (2018)]	***Echinotermes***
–	EVA armature pads raspy, truncated [see Bourguignon et al. (2016a)]	***Amplucrutermes***

## ﻿Discussion

**Figure 12.**
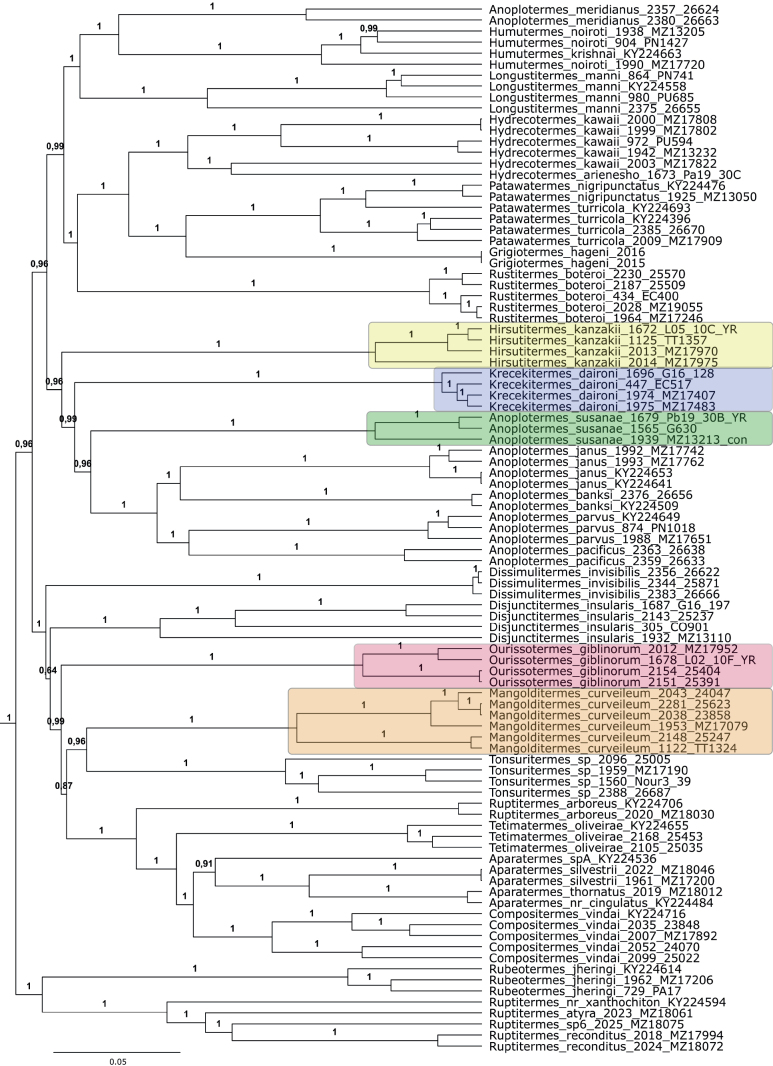
Bayesian phylogenetic tree of the New World Apicotermitinae using the complete mitogenome. New taxa are highlighted, and outgroups are not shown. Branch support is posterior probability.

The systematic and taxonomic delineation of Neotropical soldierless termites has been implemented by various methods (Bourguignon et al. 2013). For example, molecular phylogenetics and DNA barcoding allow the separation of morphologically cryptic taxa, but low branch supports for larger clades make phylogenetic reconstructions uncertain (Bourguignon et al. 2010, 2016b, Scheffrahn et al. 2017, Castro et al. 2018, 2020). Here, we provided a mitogenome phylogeny including most genera of Neotropical Apicotermitinae. Both our analyses (ML and BI) recovered the Neotropical Apicotermitinae as a well-supported monophyletic group (Fig. 12 and Suppl. material 2). The results of both analyses were similar, with the most notable exception being the position of *Hirsutitermes* gen. nov., *Rubeotermes*, and *Ruptitermes*. The new genus *Hirsutitermes* was recovered as the sister group of a clade composed of *Krecekitermes* and *Anoplotermes* in the BI (Fig. 12), but as the sister group to all New World Apicotermitinae in the ML (Suppl. material 2). In the BI, a clade composed by *Rubeotermes* and *Ruptitermes* was recovered as sister group to all New World Apicotermitinae (Fig. 12), while in the ML, these two genera were not recovered as a clade, with *Rubeotermes* as sister group to *Rustitermes*, and *Ruptitermes* close related to a clade composed of *Grigiotermes*, *Patawatermes* and *Hydrecotermes*. With our data, no conclusions can be made regarding the relationship of these groups.

**Figure 13.**
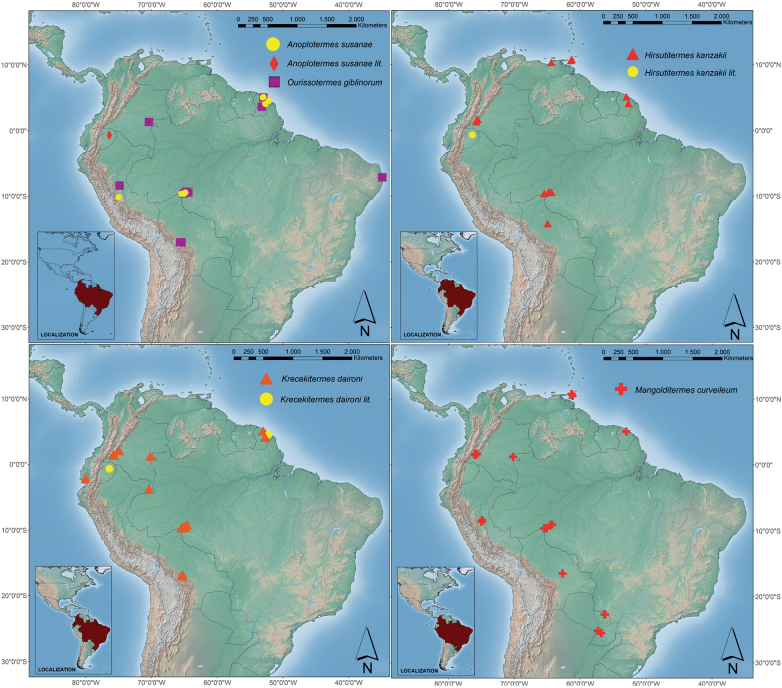
Distribution maps of the new species described in this study (lit.= literature record).

A few groups showed high branch support values and deserve a note. The genera *Grigiotermes* and *Patawatermes* (originally described as *Grigiotermes*) form a clade and share many morphological characters. If future analysis corroborates the results found here, reverting the *Patawatermes* species to the genus *Grigiotermes* may be a parsimonious taxonomic decision. A group composed of *Compositermes*, *Aparatermes*, *Tetimatermes*, and the species *Ruptitermesarboreus* seems to be a very consistent group, despite the lack of morphological synapomorphies, this clade was also recovered in recent works using the COI gene (e.g., Pinzón et al. 2019; Castro et al. 2020).

Remarkable diagnostic morphological characters in New World Apicotermitinae occur in a few genera (e.g., *Compositermes*, *Tonsuritermes*, *Tetimatermes*), but there is a lack of remarkable diagnostic characters for differentiating other genera. For example, *Anoplotermes* and *Humutermes* share a similar digestive tract, and these genera can only be supported by molecular data. This difficulty becomes even clearer in our phylogeny, where the species *Anoplotermesmeridianus* clustered with *Humutermes*, making *Anoplotermes* paraphyletic. In addition, the new species *Anoplotermessusanae* sp. nov. clustered with the *Anoplotermes* “stricto sensu”, but with low branch support. Using the barcode gene COI alone (unpublished data), this species was first hypothesized as a new genus. The inclusion of more genetic data, such as nuclear genes, will be important to corroborate our results here. Likewise, we emphasize the large number of species whose type material was not yet examined in recent studies, and synonyms can be generated in the future.

Another critical challenge occurs within species with unarmed EVA, where the characters are even more inconspicuous, making species identification more difficult. In this study, we identified morphospecies records from pictures from other studies (Bourguignon et al. 2013; Dahlsjö et al. 2020; Castro et al. 2021) for the species *A.susanae* sp. nov., *Hirsutitermeskanzakii* sp. nov., and *Krecekitermesdaironi* sp. nov., but not for *M.curveileum* sp. nov., even though this was the species with the highest reported distribution. This species may have already been reported, but available EVA photographs generally do not show the cushions in great detail, or they are covered with muscular tissue that makes the internal structures difficult to see; while the well-sclerotized armature can be seen even with muscle tissue involved (Bourguignon et al. 2013; Dahlsjö et al. 2020). The identification of species using the enteric valve morphology is crucial for the reliable identification of soldierless termites, as has been done in ecological studies (Dahlsjö et al. 2020; Castro et al. 2021). However, the photographic material and description of some species do not seem to be optimal, preventing comparisons (Bourguignon et al. 2016b). The addition of diagnostic characters such as enteric valve seating (EVS), would have been able to avoid this misidentification; or the better appreciation of the scales forms on the cushions could have helped to discriminate these species, but unfortunately this character is not possible to see clearly in the *Patawatermes* original description.

Despite the large number of descriptions of new taxa of soldierless termites from the Neotropics in the last decade (Bourguignon et al. 2010, 2016a; Carrijo et al. 2015; Scheffrahn et al. 2017; Castro et al. 2018, 2020; Constantini et al. 2018, 2020; Pinzón et al. 2019), ecological studies still show the existence of an enormous taxonomic-gap within the Neotropical soldierless termites. These studies always present their species list with most of Apicotermitinae only as morphotypes, either for the lack of identification skills or the large number of new taxa yet to be described (Casalla and Korb 2019; da Silva et al. 2019; Dahlsjö et al. 2020; Duran-Bautista et al. 2020a, b; De Azevedo et al. 2021; Cunha et al. 2021). Although it could be assumed that the description of new taxa could be geographically limited, as evidenced for other Neotropical termites (Constantino et al. 2006; Rocha and Cancello 2009; Rocha et al. 2012; Carrijo et al. 2020), a large number of soldierless taxa have a wide distribution and high abundance, such as *Humutermes* spp, *Hydrecotermes* spp, *Patawatermes* spp, *Disjunctitermesinsularis*, *Grigiotermeshageni*, *Rustitermesboteroi*, and *Rubeotermesjheringi* (Bourguignon et al. 2016b, Scheffrahn et al. 2017; Castro et al. 2020). In addition, species such as *Patawatermesturricola*, *Disjunctitermesinsularis*, and *Rustitermesboteroi* have been identified as indicators in degraded soils of Andean-Amazon piedmont, where they are abundant (Castro et al. 2021), highlighting the role of these termites in soil recovery and other ecosystem services.

The soldierless termites present not only a taxonomic gap (Linnean shortfall), but also a geographical gap (Wallacean shortfall) (Whittaker et al. 2005; Bini et al. 2006). Since most samples from museums and collections are yet to be studied and there is a lack of inventories in many areas, the geographic gap for these termites is even greater than for other groups. This is also true in the other parts of the world, where the taxonomic gap is not so deep because of the older available literature (Grassé and Noirot 1954; Sands 1972), but the geographical gap still exists due the lack of researchers for a long period (Romero Arias et al. 2021).

The key that we provide can serve as a support tool for the confirmation of identification of genera that are already described; however, as we discuss here, there is many species not yet described. It is expected that progress in the description of soldierless taxa will highlight the importance of these groups in ecological studies and once ecological studies start naming the soldierless termites, the Wallacean shortfall of these termites will also diminish.

## Supplementary Material

XML Treatment for Anoplotermes susanae

XML Treatment for Hirsutitermes

XML Treatment for Hirsutitermes kanzakii

XML Treatment for Krecekitermes

XML Treatment for Krecekitermes daironi

XML Treatment for Mangolditermes

XML Treatment for Mangolditermes curveileum

XML Treatment for Ourissotermes

XML Treatment for Ourissotermes giblinorum

## Footnotes

The EVA of Ruptitermes bandeirai is armed with fewer than 13 spines; however, we hypothesize that this species belongs to another genus due its gut and EVA morphology (Acioli and Constantino 2015).

## ﻿Additional information

### Author ORCIDs

Tiago F. Carrijo https://orcid.org/0000-0001-6308-7252

Daniel Castro https://orcid.org/0000-0002-5060-6129

Menglin Wang https://orcid.org/0000-0003-2206-9503

Joice P. Constantini https://orcid.org/0000-0002-1813-9276

Thomas Bourguignon https://orcid.org/0000-0002-4035-8977

Eliana M. Cancello https://orcid.org/0000-0003-3125-6335

Yves Roisin https://orcid.org/0000-0001-6635-3552

Rudolf H. Scheffrahn https://orcid.org/0000-0002-6191-5963

### Data availability

All of the data that support the findings of this study are available in the main text or Supplementary Information.

## Supplementary materials

Supplementary material 1 Samples used in the phylogenetic analysis Data type table (word document) https://binary.pensoft.net/file/867551This dataset is made available under the Open Database License (http://opendatacommons.org/licenses/odbl/1.0/). The Open Database License (ODbL) is a license agreement intended to allow users to freely share, modify, and use this Dataset while maintaining this same freedom for others, provided that the original source and author(s) are credited.Tiago F. Carrijo, Daniel Castro, Menglin Wang, Joice P. Constantini, Thomas Bourguignon, Eliana M. Cancello, Yves Roisin, Rudolf H. Scheffrahn

Supplementary material 2 Maximum Likelihood phylogenetic tree of the New World Apicotermitinae using the complete mitogenome Data type figure (word document) https://binary.pensoft.net/file/867552This dataset is made available under the Open Database License (http://opendatacommons.org/licenses/odbl/1.0/). The Open Database License (ODbL) is a license agreement intended to allow users to freely share, modify, and use this Dataset while maintaining this same freedom for others, provided that the original source and author(s) are credited.Tiago F. Carrijo, Daniel Castro, Menglin Wang, Joice P. Constantini, Thomas Bourguignon, Eliana M. Cancello, Yves Roisin, Rudolf H. Scheffrahn
