# Supplementary material for: ﻿Diminishing the taxonomic gap in the neotropical soldierless termites: descriptions of four new genera and a new Anoplotermes species (Isoptera, Termitidae, Apicotermitinae)
Source: Zookeys. 2023 Jun 22;1167:317–52. doi: 10.3897/zookeys.1167.100001 (PMC10311428; doi:10.3897/zookeys.1167.100001)
Supplement: Supplementary material 1 — Samples used in the phylogenetic analysis [file zookeys-1167-317_article-100001__-s001.docx]

Table S1. Samples used in the phylogenetic analysis.

| **Outgroups** |  | | **Count** | | **Collection Code** | | **GenBank** | | |
| --- | --- | --- | --- | --- | --- | --- | --- | --- | --- |
| *Acidnotermes* | *praus* | |  | |  | | KY224646 | | |
| *Alyscotermes* | *kilamandjaricus* | |  | |  | | KY224395 | | |
| *Amitermes* | *meridionalis* | |  | |  | | KY224487 | | |
| *Astalotermes* | *murcus* | |  | |  | | KY224676 | | |
| *Ateuchotermes* | *sentosus* | |  | |  | | KY224674 | | |
| *Cavitermes* | *tuberosus* | |  | |  | | KY224568 | | |
| *Coatitermes* | *kartaboensis* | |  | |  | | KY224708 | | |
| *Cubitermes* | *fungifaber* | |  | |  | | KP026265 | | |
| *Duplidentitermes* | *furcatidens* | |  | |  | | KP026271 | | |
| *Ephelotermes* | *melachoma* | |  | |  | | KY224392 | | |
| *Euhamitermes* | *hamatus* | |  | |  | | KY224652 | | |
| *Euhamitermes* | spA | |  | |  | | KY224727 | | |
| *Heterotermes* | *tenuis* | |  | |  | | KU925233 | | |
| *Hospitalitermes* | *hospitalis* | |  | |  | | KY224508 | | |
| *Jugositermes* | *tuberculatus* | |  | |  | | KY224532 | | |
| *Labiotermes* | *labralis* | |  | |  | | KP026292 | | |
| *Leucopitermes* | *leucops* | |  | |  | | KY224452 | | |
| *Macrotermes* | *subhyalinus* | |  | |  | | KY224559 | | |
| *Microcerotermes* | *crassus* | |  | |  | | KY224445 | | |
| *Mirocapritermes* | *connectens* | |  | |  | | KY224537 | | |
| *Nasutitermes* | *macrocephalus* | |  | |  | | KY224724 | | |
| *Neocapritermes* | *taracua* | |  | |  | | KY224527 | | |
| *Postsubulitermes* | *parviconstrictus* | |  | |  | | KP026268 | | |
| *Silvestritermes* | *heyeri* | |  | |  | | KP026288 | | |
| *Termes* | *fatalis* | |  | |  | | KY224726 | | |
|  |  | |  | |  | |  | | |
| **New World Apicotermitinae** | | | | **Count** | | **Collection Code** | | **GenBank** |  |
| Anoplotermes | | banksi | | 2376 | | MZ26656 | | OQ999258 |  |
| Anoplotermes | | banksi | |  | |  | | KY224509 |  |
| Anoplotermes | | janus | | 1992 | | MZ17742 | | OQ999259 |  |
| Anoplotermes | | janus | | 1993 | | MZ17762 | | OQ999260 |  |
| Anoplotermes | | janus | |  | |  | | KY224641 |  |
| Anoplotermes | | janus | |  | |  | | KY224653 |  |
| Anoplotermes | | meridianus | | 2357 | | MZ26624 | | OQ999265 |  |
| Anoplotermes | | meridianus | | 2380 | | MZ26663 | | OQ999266 |  |
| Anoplotermes | | pacificus | | 2359 | | MZ26633 | | OQ999261 |  |
| Anoplotermes | | pacificus | | 2363 | | MZ26638 | | OQ999262 |  |
| Anoplotermes | | parvus | | 1988 | | MZ17651 | | OQ999264 |  |
| Anoplotermes | | parvus | | 874 | | PN1018 | | OQ999263 |  |
| Anoplotermes | | parvus | |  | |  | | KY224649 |  |
| Anoplotermes | | susanae | | 1565 | | G630 | | OQ999300 |  |
| Anoplotermes | | susanae | | 1939 | | MZ13213 | | OQ999316 |  |
| Anoplotermes | | susanae | | 1679 | | Pb19.30B.YR | | OQ999325 |  |
| Aparatermes | | nr.cingulatus | |  | |  | | KY224484 |  |
| Aparatermes | | silvestrii | | 1961 | | MZ17200 | | OQ999275 |  |
| Aparatermes | | silvestrii | | 2022 | | MZ18046 | | OQ999276 |  |
| Aparatermes | | spA | |  | |  | | KY224536 |  |
| Aparatermes | | thornatus | | 2019 | | MZ18012 | | OQ999274 |  |
| Compositermes | | vindai | | 2007 | | MZ17892 | | OQ999278 |  |
| Compositermes | | vindai | | 2035 | | MZ23848 | | OQ999277 |  |
| Compositermes | | vindai | | 2052 | | MZ24070 | | OQ999279 |  |
| Compositermes | | vindai | | 2099 | | MZ25022 | | OQ999280 |  |
| Compositermes | | vindai | |  | |  | | KY224716 |  |
| Disjuncitermes | | insularis | | 305 | | CO901 | | OQ999270 |  |
| Disjuncitermes | | insularis | | 1687 | | G16.197 | | OQ999271 |  |
| Disjuncitermes | | insularis | | 2143 | | MZ25237 | | OQ999272 |  |
| Disjunctitermes | | insularis | | 1932 | | MZ13110 | | OQ999273 |  |
| Dissimulitermes | | invisibilis | | 2344 | | MZ25871 | | OQ999322 |  |
| Dissimulitermes | | invisibilis | | 2356 | | MZ26622 | | OQ999323 |  |
| Dissimulitermes | | invisibilis | | 2383 | | MZ26666 | | OQ999324 |  |
| Grigiotermes | | hageni | | 2015 | |  | | OQ999247 |  |
| Grigiotermes | | hageni | | 2016 | |  | | OQ999248 |  |
| Hirsutitermes | | kanzakii | | 1672 | | L05.10C.YR | | OQ999297 |  |
| Hirsutitermes | | kanzakii | | 2013 | | MZ17970 | | OQ999298 |  |
| Hirsutitermes | | kanzakii | | 2014 | | MZ17975 | | OQ999299 |  |
| Hirsutitermes | | kanzakii | | 1125 | | TT1357 | | OQ999296 |  |
| Humutermes | | krishnai | |  | |  | | KY224663 |  |
| Humutermes | | noiroti | | 1938 | | MZ13205 | | OQ999301 |  |
| Humutermes | | noiroti | | 1990 | | MZ17720 | | OQ999303 |  |
| Humutermes | | noiroti | | 904 | | PN1427 | | OQ999302 |  |
| Hydrecotermes | | arienesho | | 1673 | | Pa19.30C | | OQ999252 |  |
| Hydrecotermes | | kawaii | | 1942 | | MZ13232 | | OQ999254 |  |
| Hydrecotermes | | kawaii | | 1999 | | MZ17802 | | OQ999255 |  |
| Hydrecotermes | | kawaii | | 2000 | | MZ17808 | | OQ999256 |  |
| Hydrecotermes | | kawaii | | 2003 | | MZ17822 | | OQ999257 |  |
| Hydrecotermes | | kawaii | | 972 | | PU594 | | OQ999253 |  |
| Krecekitermes | | daironi | | 447 | | EC517 | | OQ999283 |  |
| Krecekitermes | | daironi | | 1696 | | G16.128 | | OQ999285 |  |
| Krecekitermes | | daironi | | 1974 | | MZ17407 | | OQ999286 |  |
| Krecekitermes | | daironi | | 1975 | | MZ17483 | | OQ999284 |  |
| Longustitermes | | manni | | 2375 | | MZ26655 | | OQ999269 |  |
| Longustitermes | | manni | | 864 | | PN741 | | OQ999267 |  |
| Longustitermes | | manni | | 980 | | PU685 | | OQ999268 |  |
| Longustitermes | | manni | |  | |  | | KY224558 |  |
| Mangolditermes | | curveileum | | 1953 | | MZ17079 | | OQ999312 |  |
| Mangolditermes | | curveileum | | 2038 | | MZ23858 | | OQ999314 |  |
| Mangolditermes | | curveileum | | 2043 | | MZ24047 | | OQ999313 |  |
| Mangolditermes | | curveileum | | 2148 | | MZ25247 | | OQ999311 |  |
| Mangolditermes | | curveileum | | 2281 | | MZ25623 | | OQ999315 |  |
| Mangolditermes | | curveileum | | 1122 | | TT1324 | | OQ999310 |  |
| Ourissotermes | | giblinorum | | 1678 | | L02.10F.YR | | OQ999287 |  |
| Ourissotermes | | giblinorum | | 2012 | | MZ17952 | | OQ999288 |  |
| Ourissotermes | | giblinorum | | 2151 | | MZ25391 | | OQ999289 |  |
| Ourissotermes | | giblinorum | | 2154 | | MZ25404 | | OQ999290 |  |
| Patawatermes | | nigripunctatus | | 1925 | | MZ13050 | | OQ999249 |  |
| Patawatermes | | nigripunctatus | |  | |  | | KY224476 |  |
| Patawatermes | | spA | |  | |  | | KY224396 |  |
| Patawatermes | | turricola | | 2009 | | MZ17909 | | OQ999250 |  |
| Patawatermes | | turricola | | 2385 | | MZ26670 | | OQ999251 |  |
| Patawatermes | | turricola | |  | |  | | KY224693 |  |
| Rubeotermes | | jheringi | | 1962 | | MZ17206 | | OQ999309 |  |
| Rubeotermes | | jheringi | | 729 | | PA17 | | OQ999308 |  |
| Rubeotermes | | jheringi | |  | |  | | KY224614 |  |
| Ruptitermes | | arboreus | | 2020 | | MZ18030 | | OQ999317 |  |
| Ruptitermes | | arboreus | |  | |  | | KY224706 |  |
| Ruptitermes | | atyra | | 2023 | | MZ18061 | | OQ999304 |  |
| Ruptitermes | | nr.xanthochiton | |  | |  | | KY224594 |  |
| Ruptitermes | | reconditus | | 2018 | | MZ17994 | | OQ999305 |  |
| Ruptitermes | | reconditus | | 2024 | | MZ18072 | | OQ999306 |  |
| Ruptitermes | | sp6 | | 2025 | | MZ18075 | | OQ999307 |  |
| Rustitermes | | boteroi | | 434 | | EC400 | | OQ999291 |  |
| Rustitermes | | boteroi | | 1964 | | MZ17246 | | OQ999292 |  |
| Rustitermes | | boteroi | | 2028 | | MZ19055 | | OQ999293 |  |
| Rustitermes | | boteroi | | 2187 | | MZ25509 | | OQ999294 |  |
| Rustitermes | | boteroi | | 2230 | | MZ25570 | | OQ999295 |  |
| Tetimatermes | | oliveirae | | 2105 | | MZ25035 | | OQ999281 |  |
| Tetimatermes | | oliveirae | | 2168 | | MZ25453 | | OQ999282 |  |
| Tetimatermes | | oliveirae | |  | |  | | KY224655 |  |
| Tonsuritermes | | sp | | 1959 | | MZ17190 | | OQ999319 |  |
| Tonsuritermes | | sp | | 2096 | | MZ25005 | | OQ999320 |  |
| Tonsuritermes | | sp | | 2388 | | MZ26687 | | OQ999321 |  |
| Tonsuritermes | | sp | | 1560 | | Nour3.39 | | OQ999318 |  |
|  |  | |  | |  | |  | | |
|  |  | |  | |  | |  | | |
