# Supplementary material for: ﻿Diminishing the taxonomic gap in the neotropical soldierless termites: descriptions of four new genera and a new Anoplotermes species (Isoptera, Termitidae, Apicotermitinae)
Source: Zookeys. 2023 Jun 22;1167:317–52. doi: 10.3897/zookeys.1167.100001 (PMC10311428; doi:10.3897/zookeys.1167.100001)
Supplement: Supplementary material 2 — Maximum Likelihood phylogenetic tree of the New World Apicotermitinae using the complete mitogenome [file zookeys-1167-317_article-100001__-s002.docx]

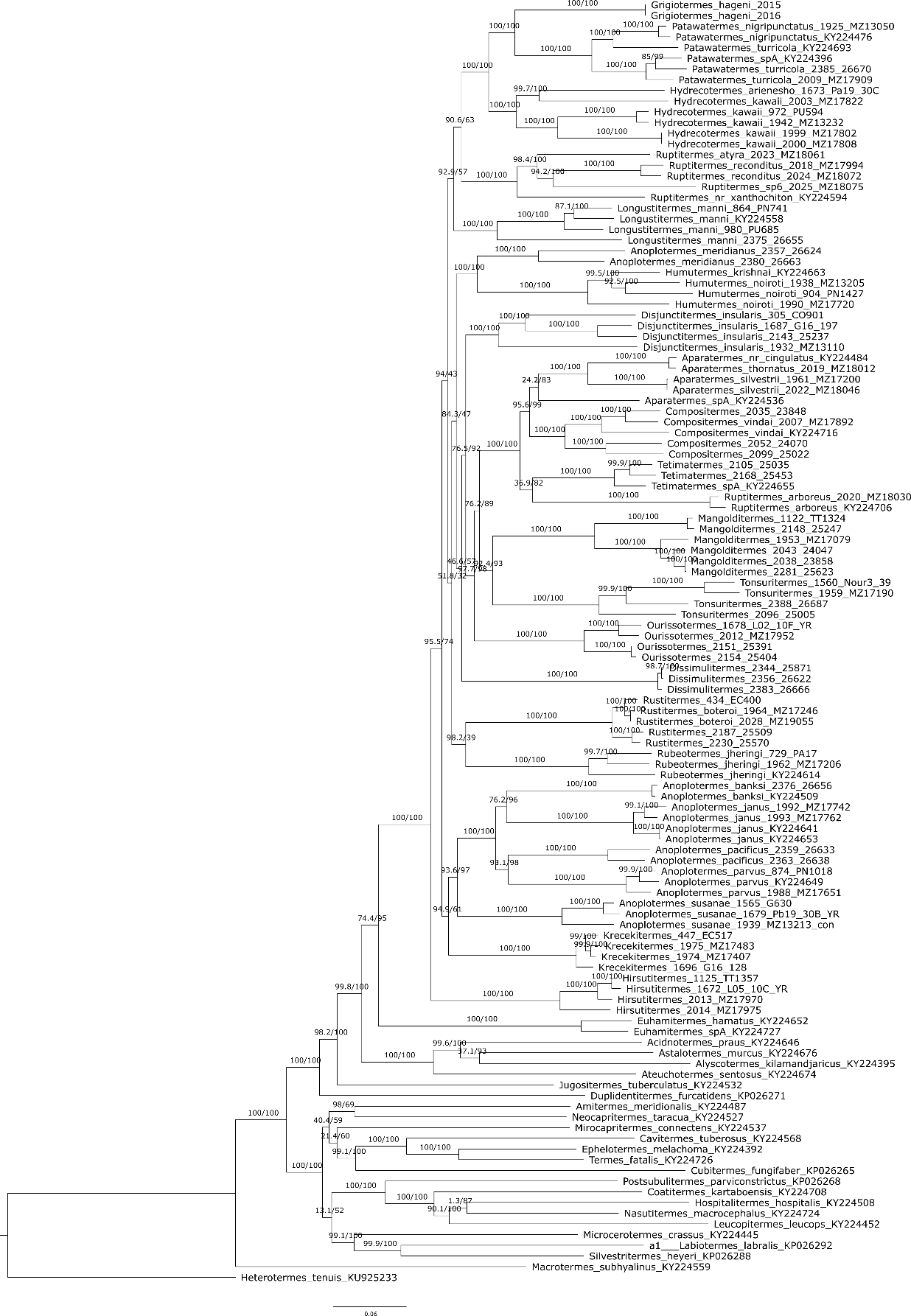


Figure S1. Maximum Likelihood phylogenetic tree of the New World Apicotermitinae using the complete mitogenome. Branch support is posterior probability. Numbers in parentheses are SH-aLRT support (%) / ultrafast bootstrap support (%).
